# Supplementary material for: Synthesis of Non-Aromatic Pyrroles Based on the Reaction of Carbonyl Derivatives of Acetylene with 3,3-Diaminoacrylonitriles
Source: Molecules. 2023 Apr 19;28(8):3576. doi: 10.3390/molecules28083576 (PMC10141133; doi:10.3390/molecules28083576)

Supplementary Materials

Synthesis of Non-Aromatic Pyrroles Based on the Reaction of Carbonyl Derivatives of Acetylene with 3,3-Diaminoacrylonitriles

Pavel S. Silaichev, Lidia N. Dianova, Tetyana V. Beryozkina, Vera S. Berseneva, Andrey N. Maslivets, Vasiliy A. Bakulev

Table of Contents

NMR spectra of compounds **4a-g** S2

NMR spectra of compounds **5a-f** S11

NMR spectra of compounds **6a-g** S17

NMR spectra of compounds **7a,b** S26

Figure S1. ^{1}H NMR spectrum (DMSO-d_6) of **4a**

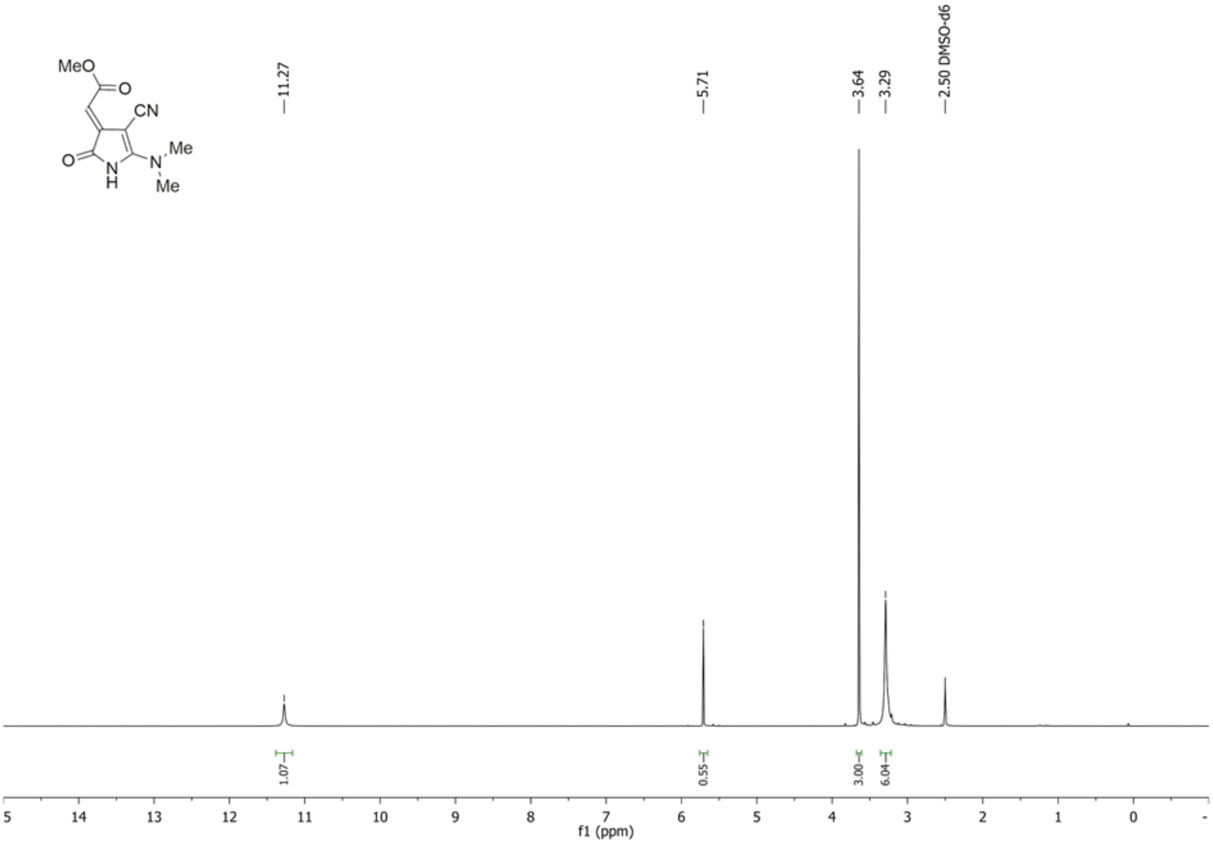

Figure S2. ^{13}C NMR spectrum (DMSO-d_6) of **4a**

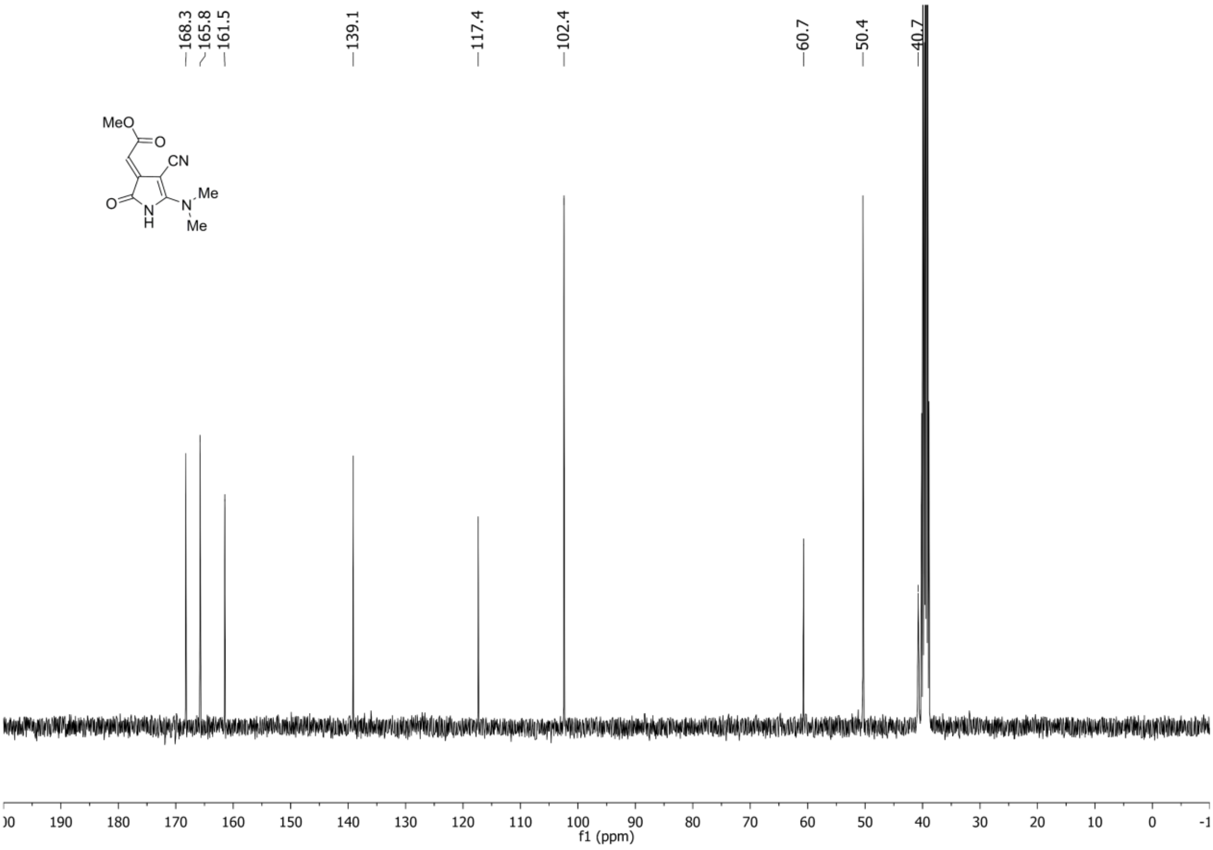

Figure S3. ^{1}H-^{13}C HSQC spectrum of **4a**

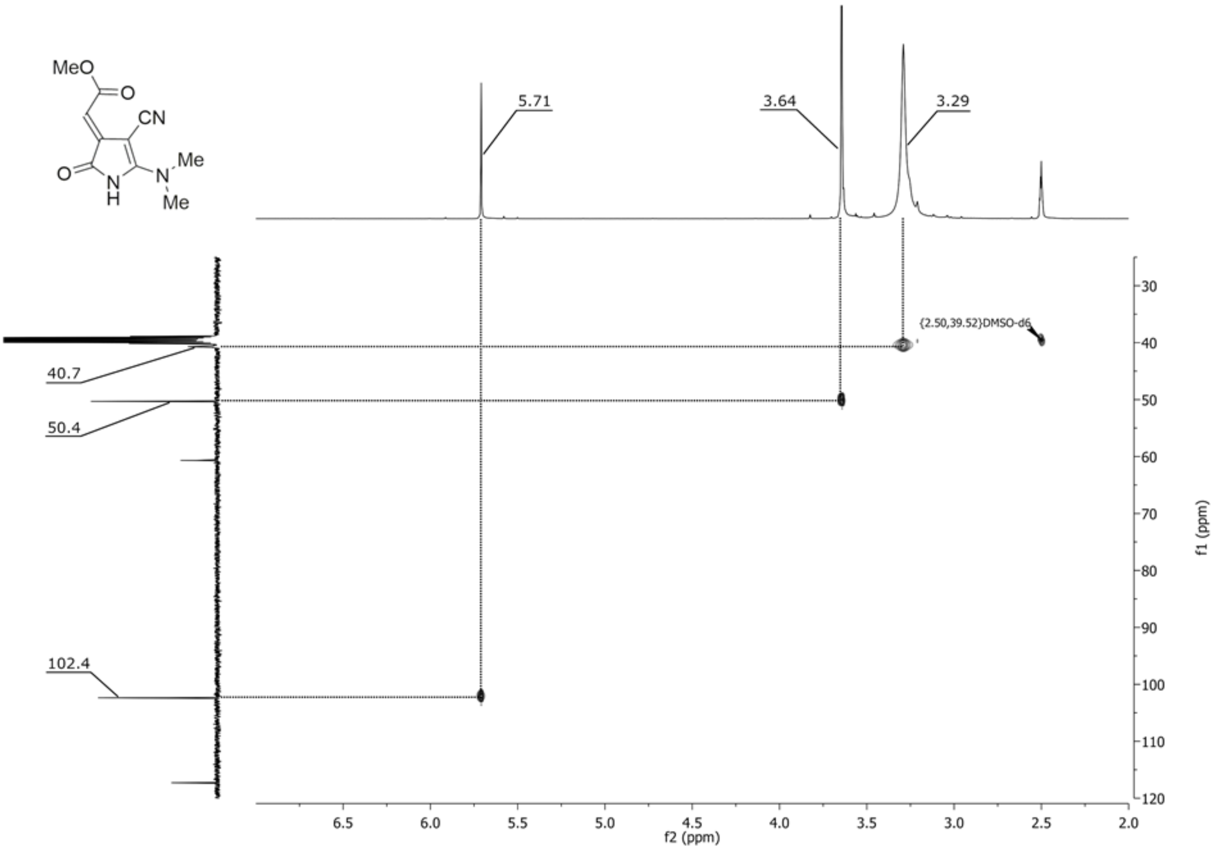

Figure S4. ^{1}H NMR spectrum (DMSO-d_6) of **4b**

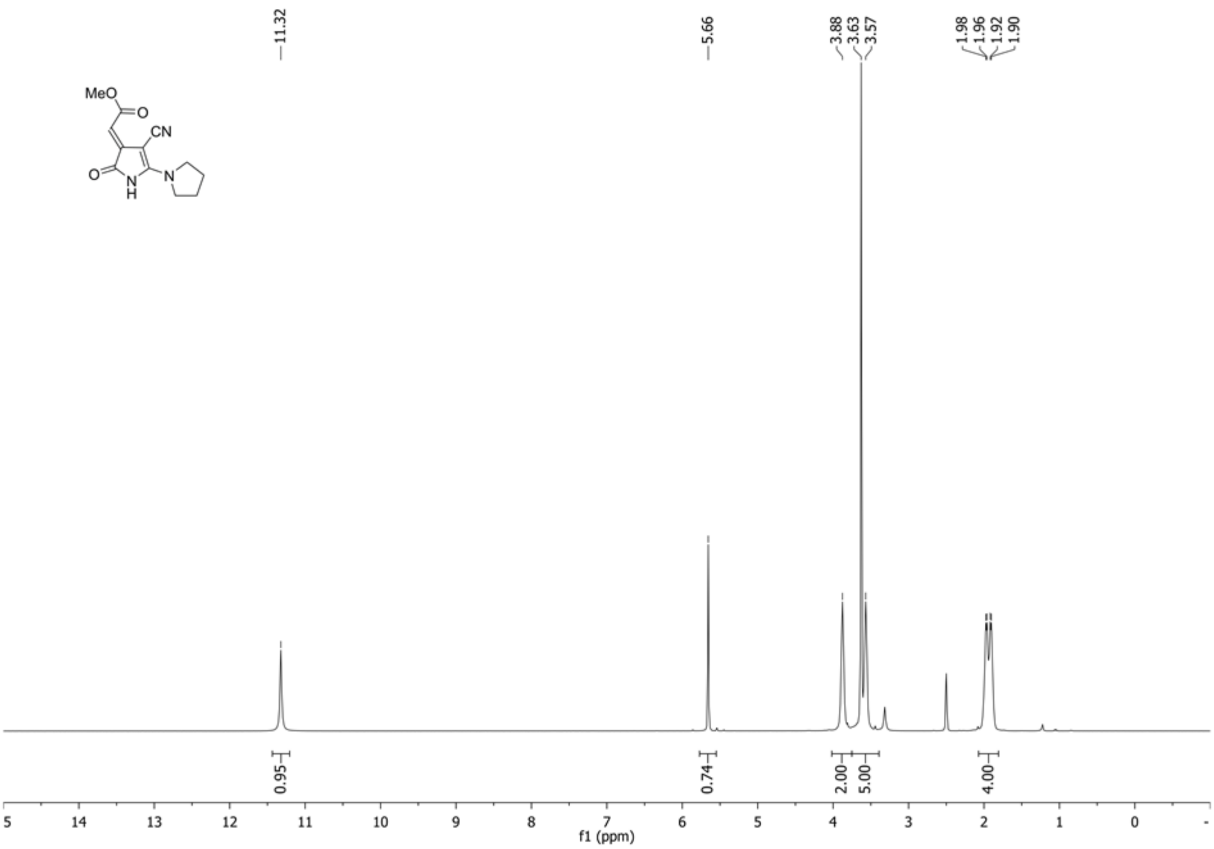

Figure S5. ^{13}C NMR spectrum (DMSO-d_6) of **4b**

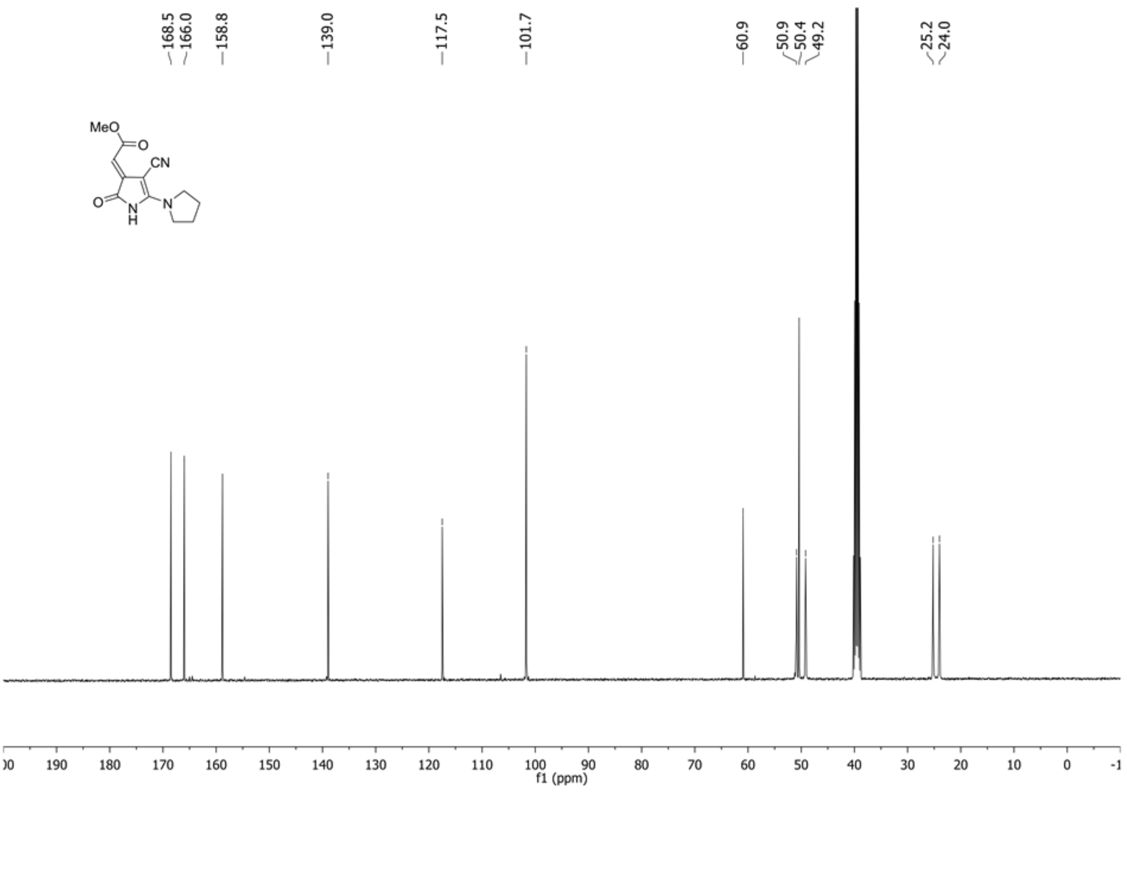

Figure S6. ^{1}H-^{13}C HSQC spectrum of **4b**

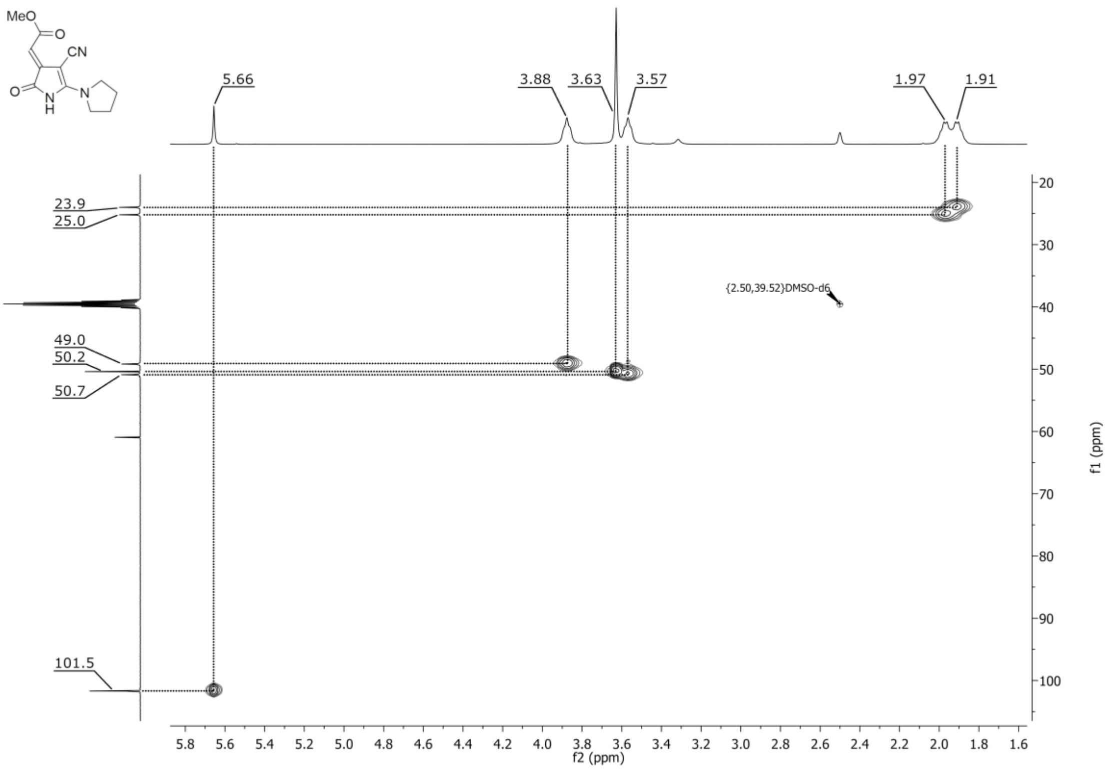

Figure S7. ^{1}H-^{13}C HMBC spectrum of **4b**

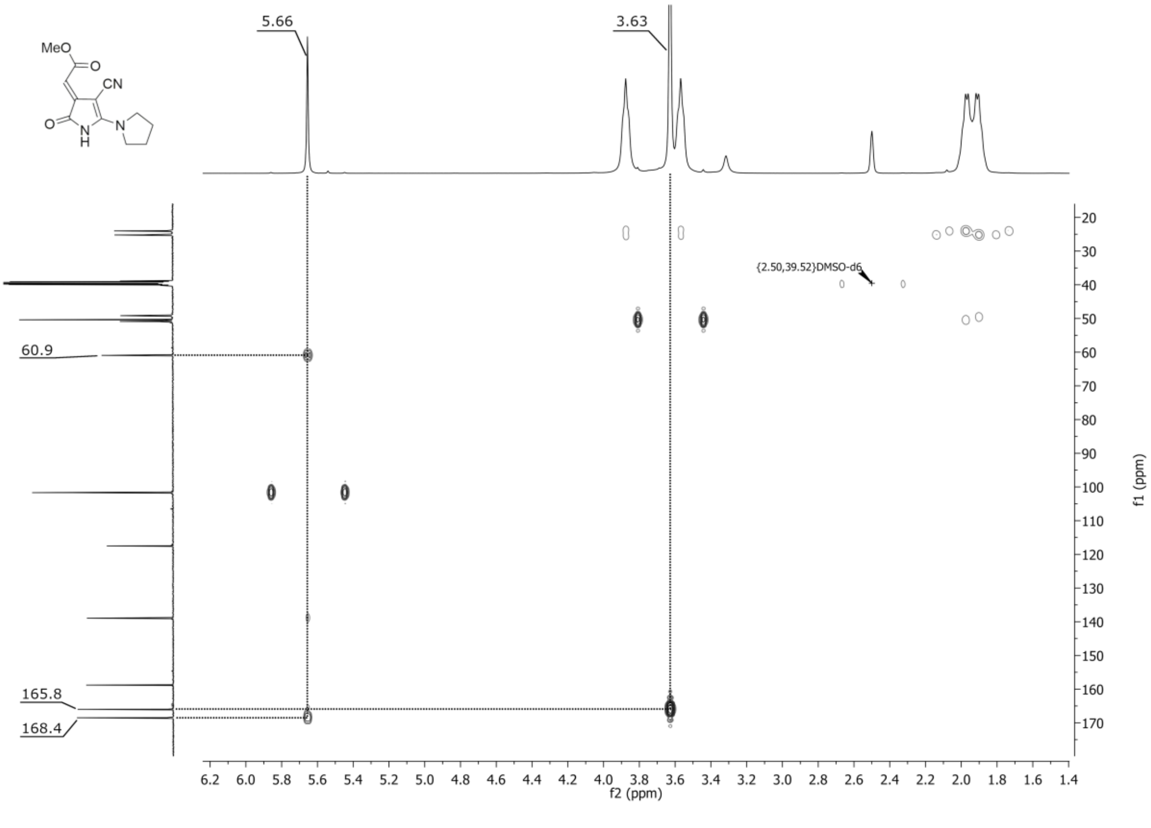

Figure S8. ^{1}H NMR spectrum (DMSO-d_6) of **4c**

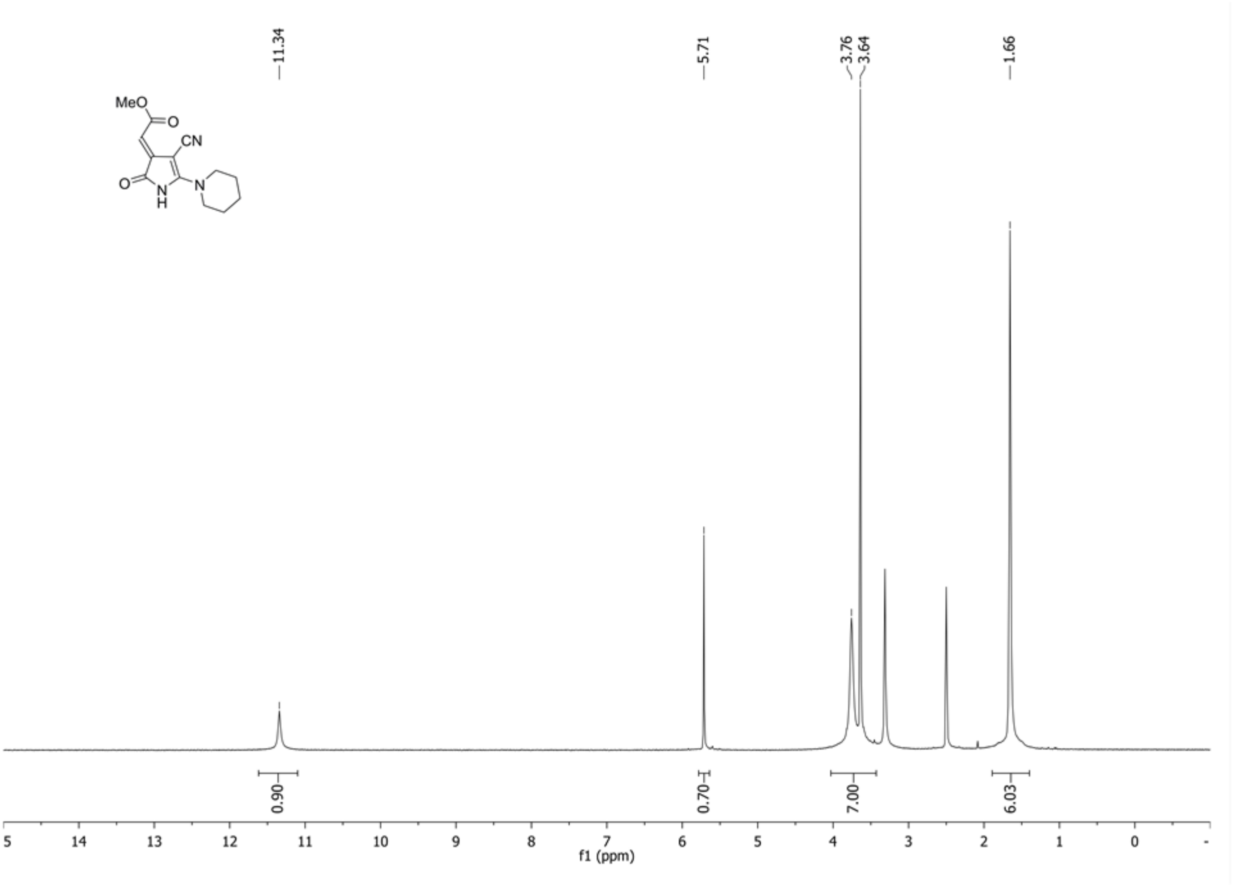

Figure S9. ^{13}C NMR spectrum (DMSO-d_6) of **4c**

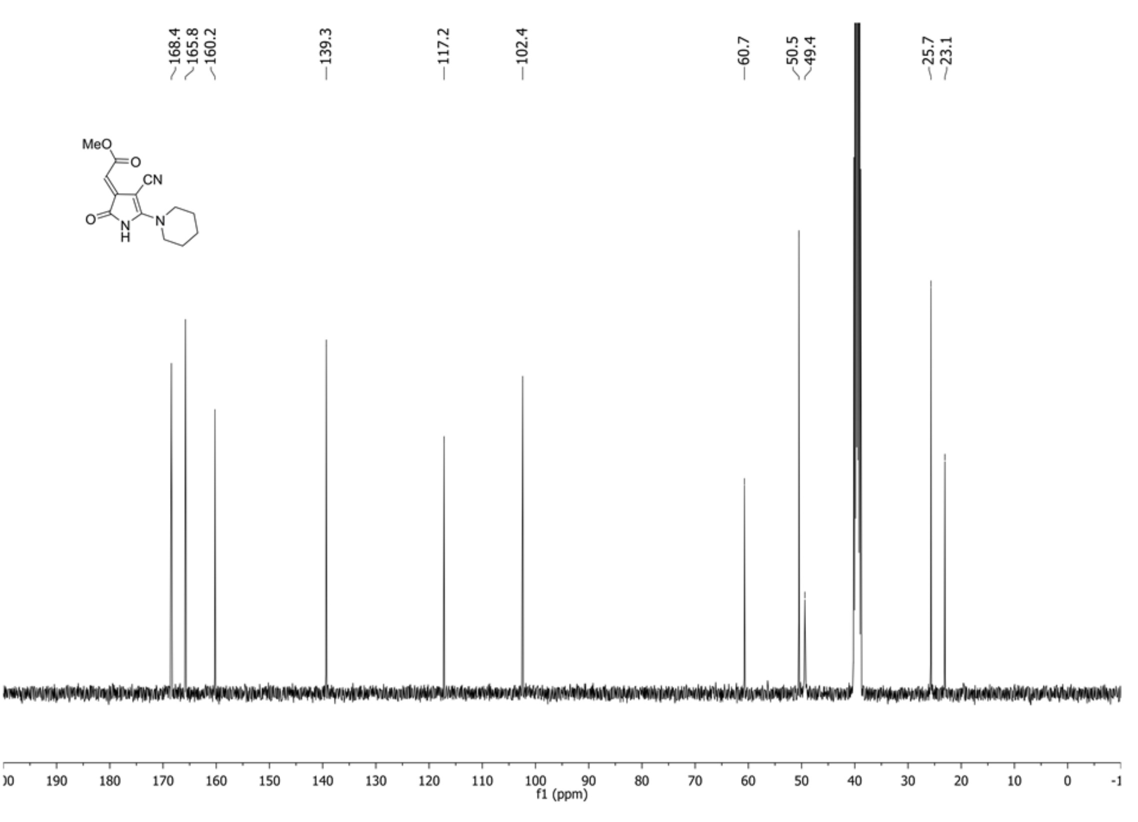

Figure S10. ^{1}H NMR spectrum (DMSO-d_6) of **4d**

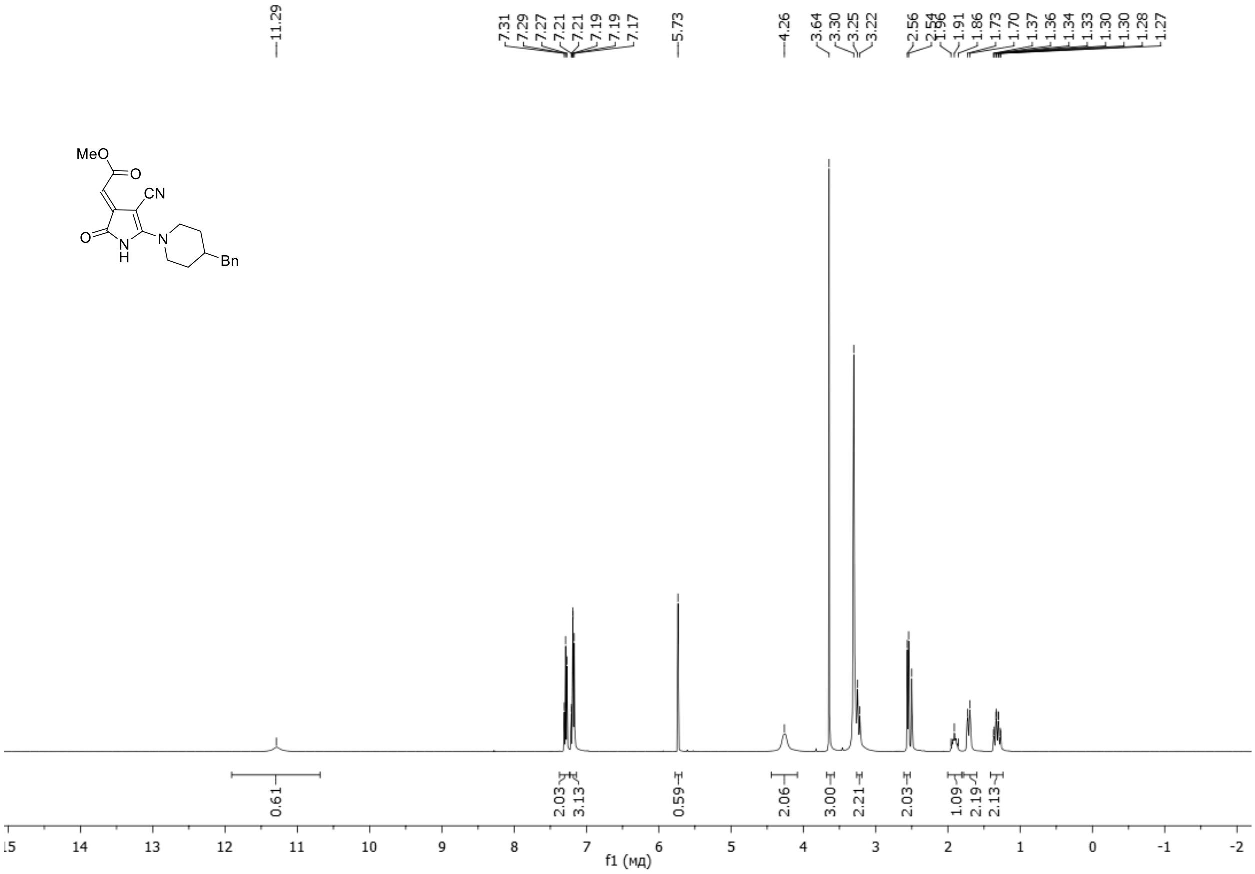

Figure S11. ^{13}C NMR spectrum (DMSO-d_6) of **4d**

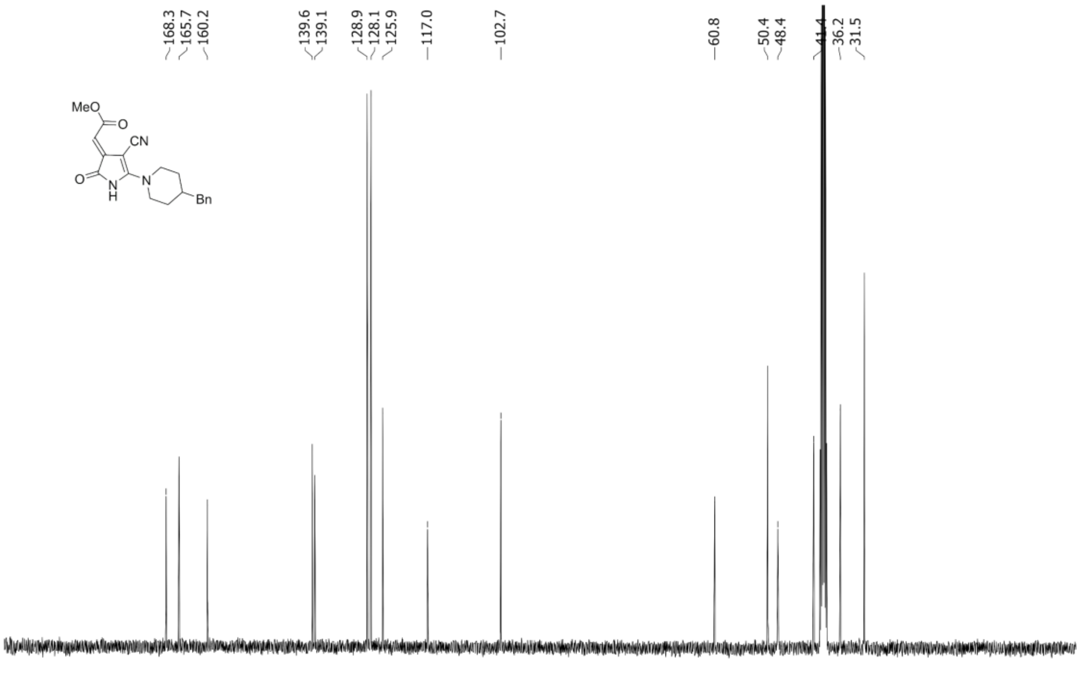

Figure S12. ^{1}H NMR spectrum (DMSO-d_6) of **4e**

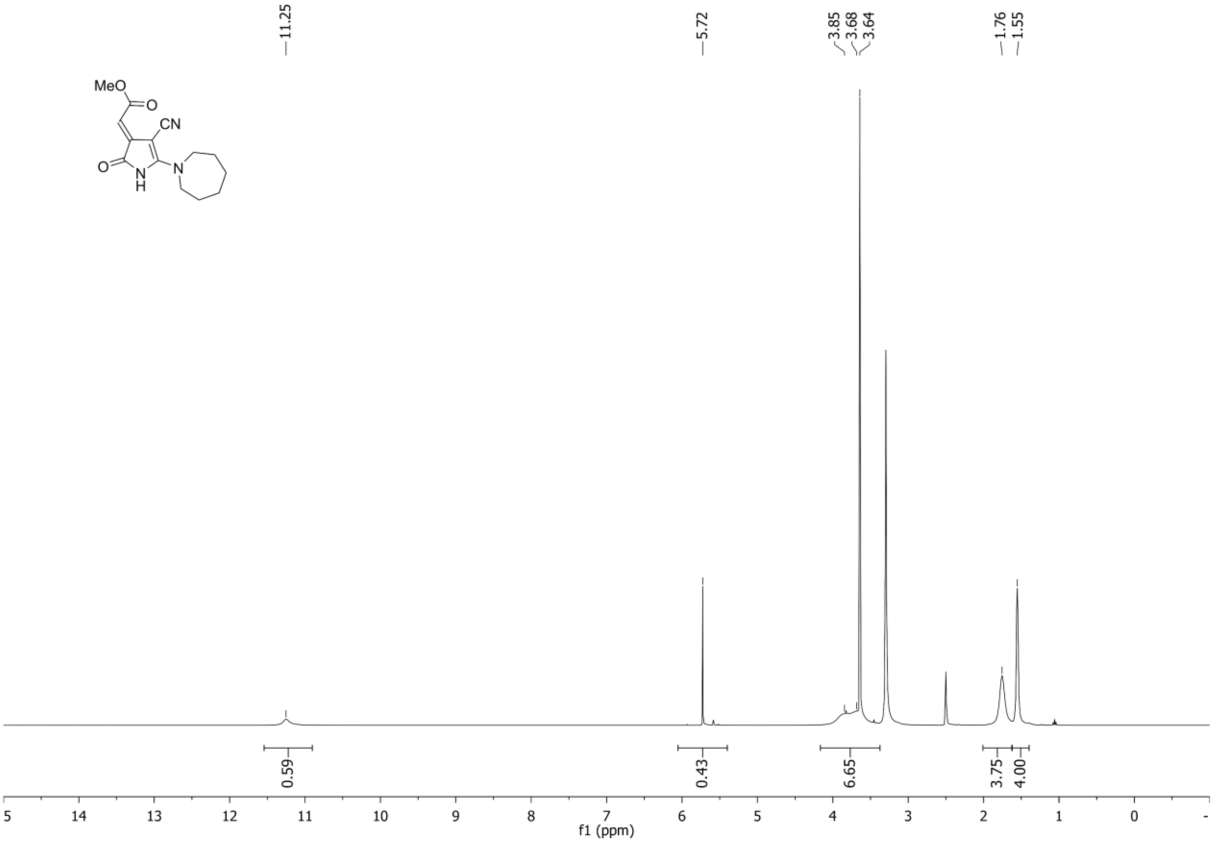

Figure S13. ^{13}C NMR spectrum (DMSO-d_6) of **4e**

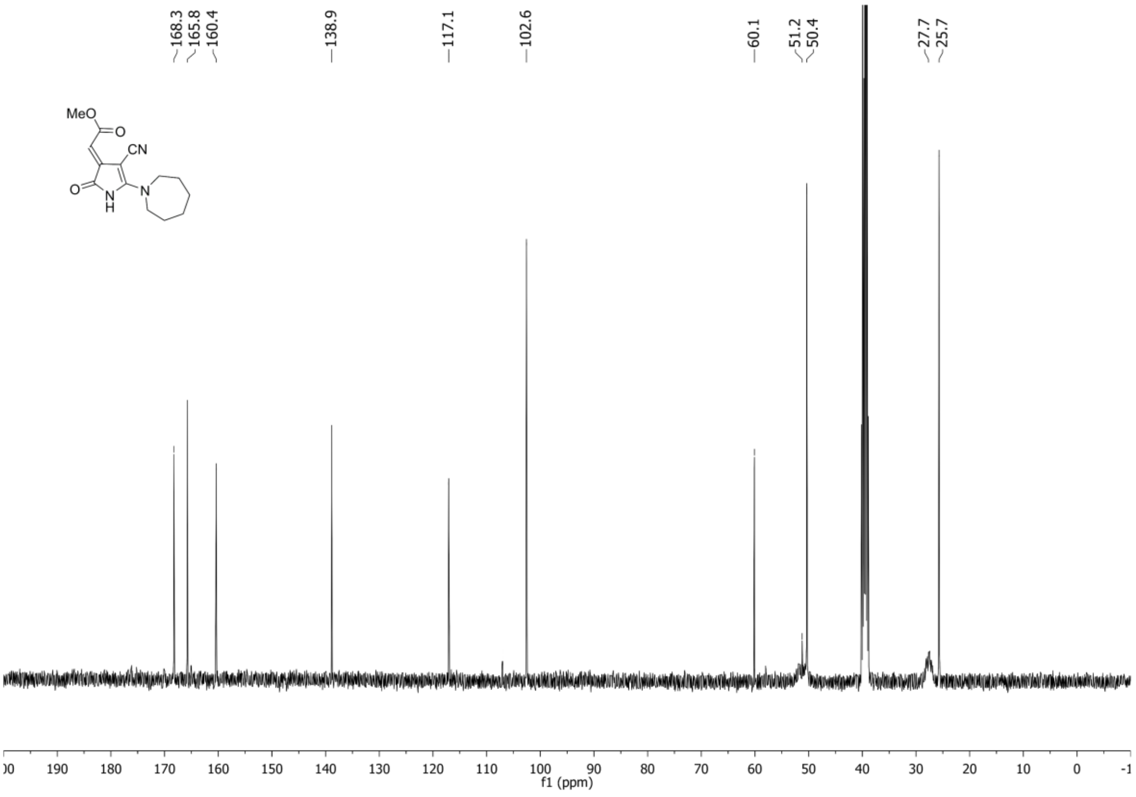

Figure S14. ^{1}H NMR spectrum (DMSO-d_6) of **4f**

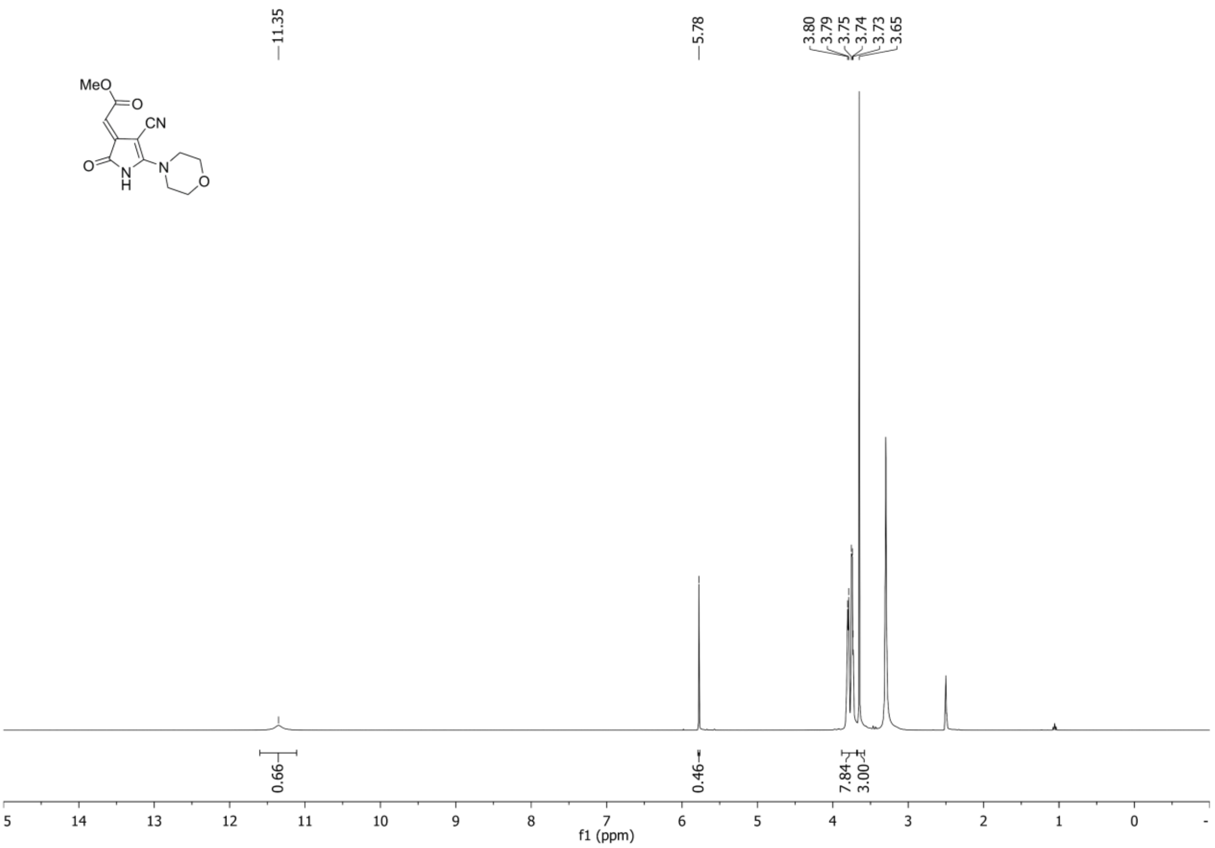

Figure S15. ^{13}C NMR spectrum (DMSO-d_6) of **4f**

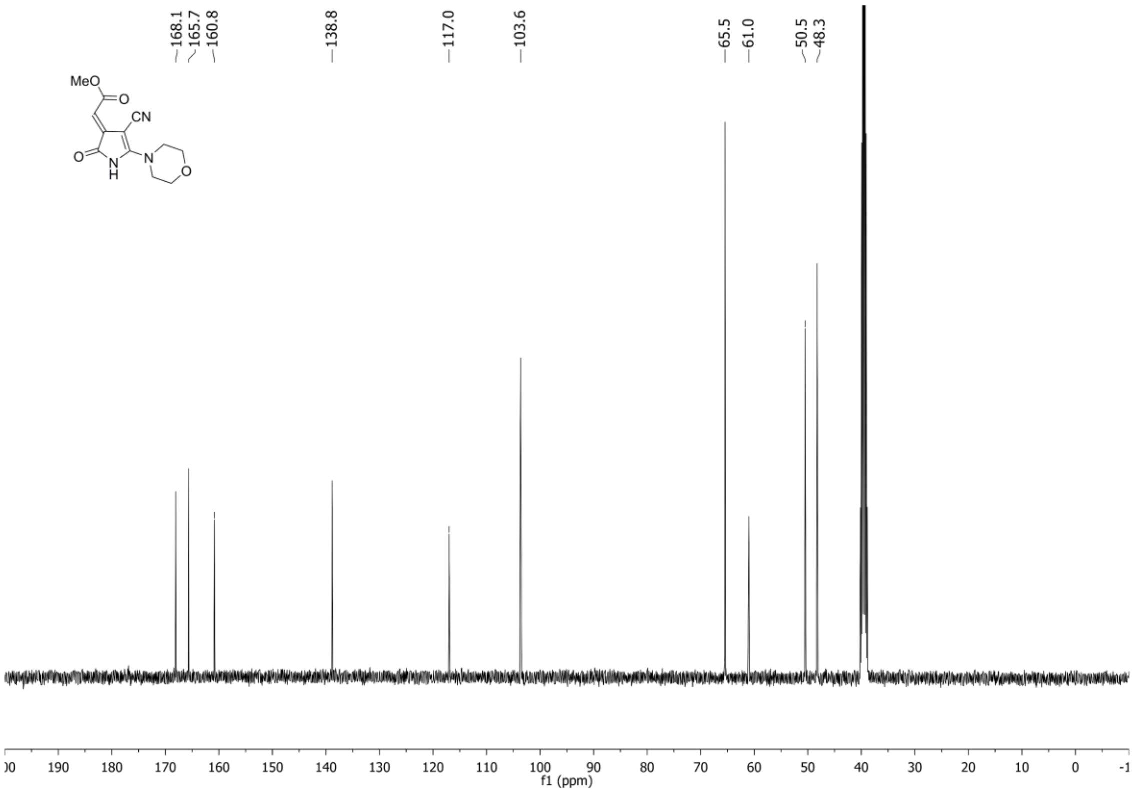

Figure S16. ^{1}H NMR spectrum (DMSO-d_6) of **4g**

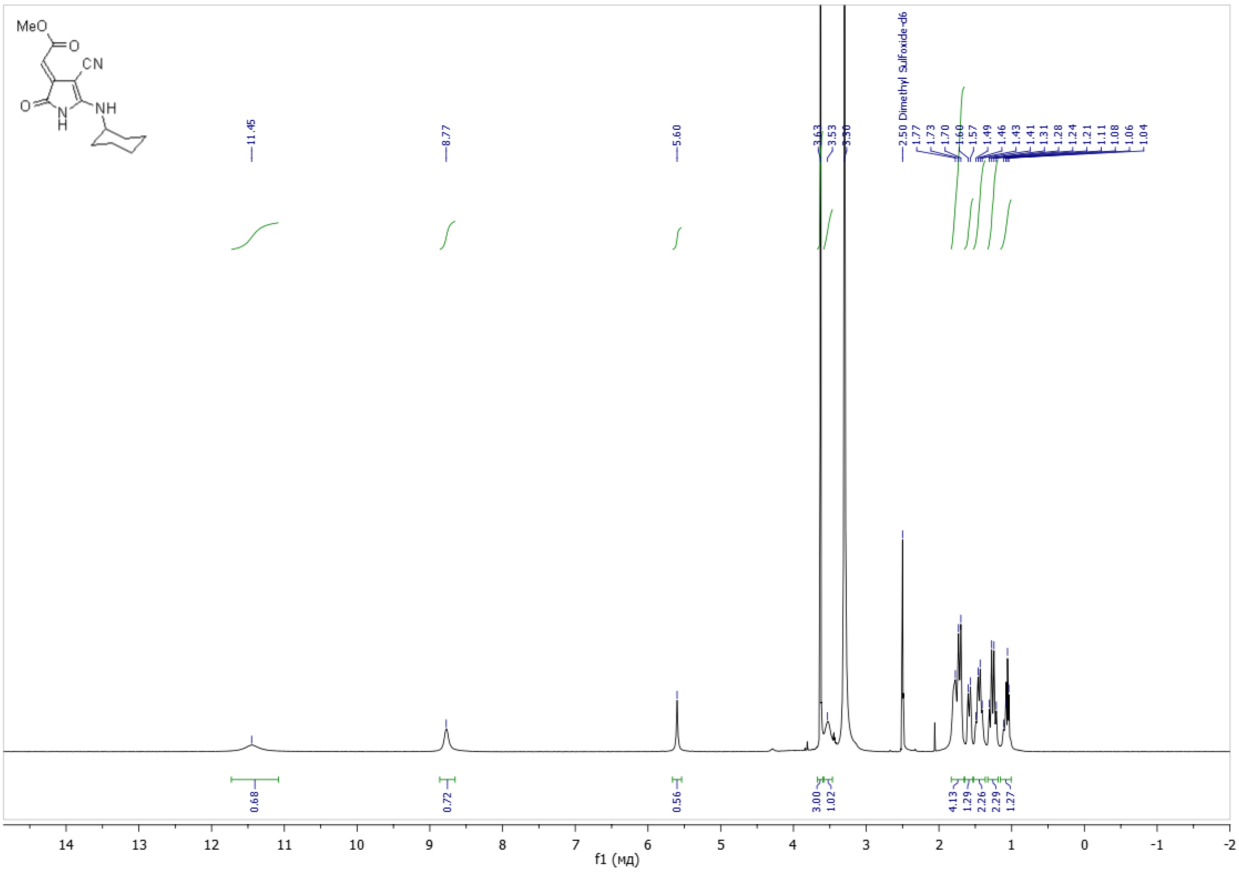

Figure S17. ^{1}H NMR spectrum (DMSO-d_6) of **4g**

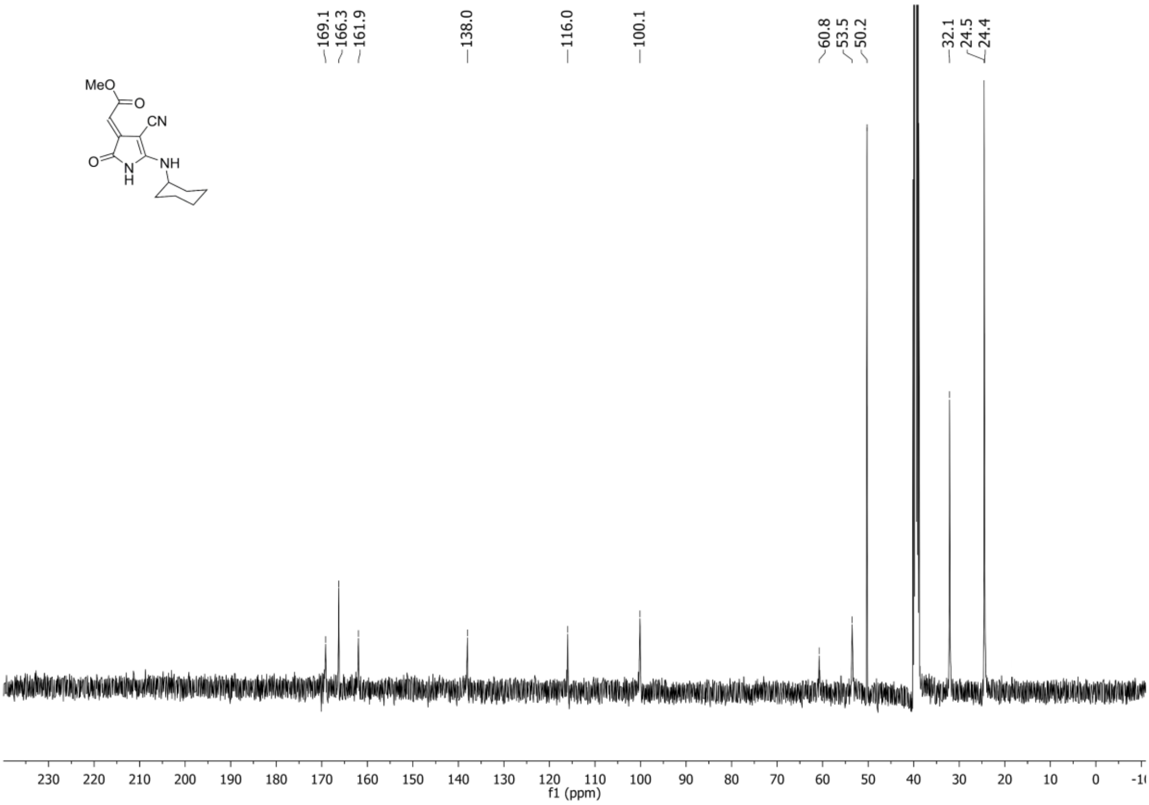

Figure S18. ^{1}H NMR spectrum (DMSO-d_6) of **5a**

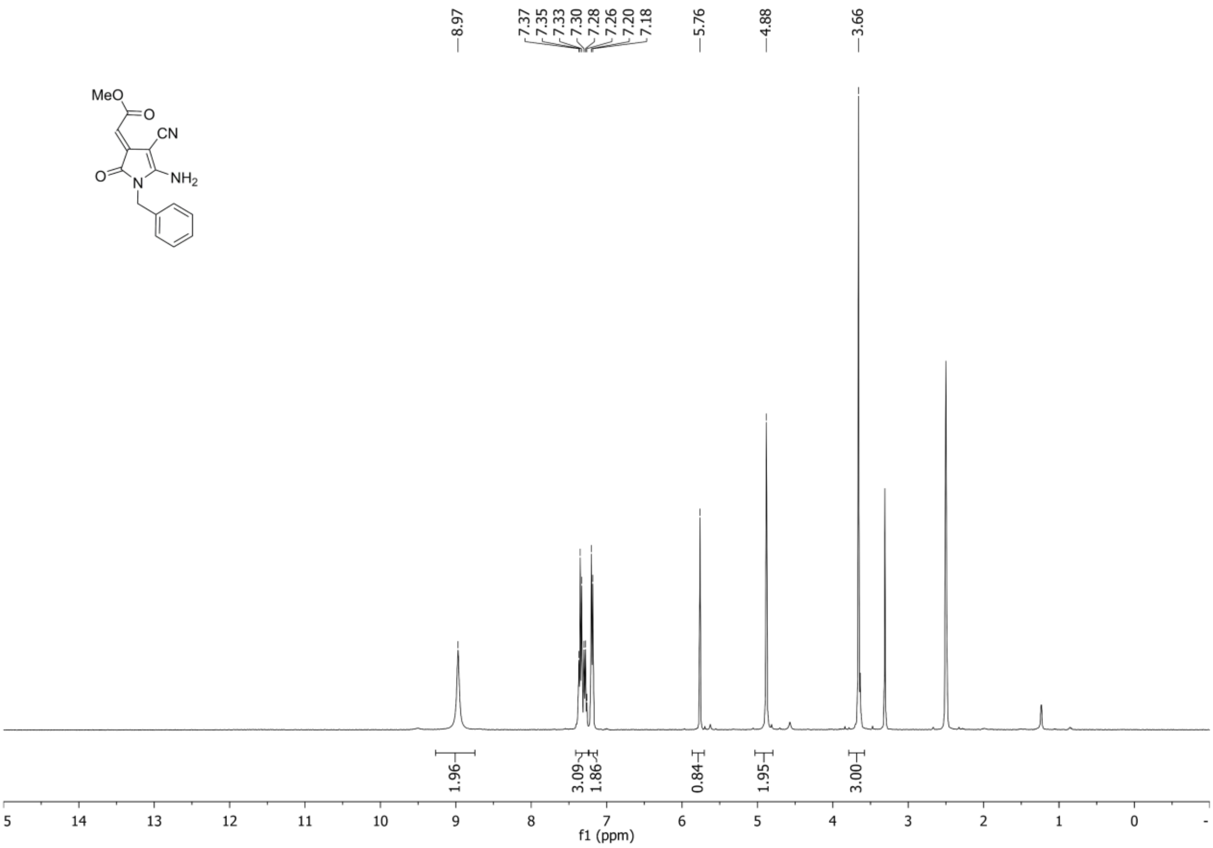

Figure S19. ^{13}C NMR spectrum (DMSO-d_6) of **5a**

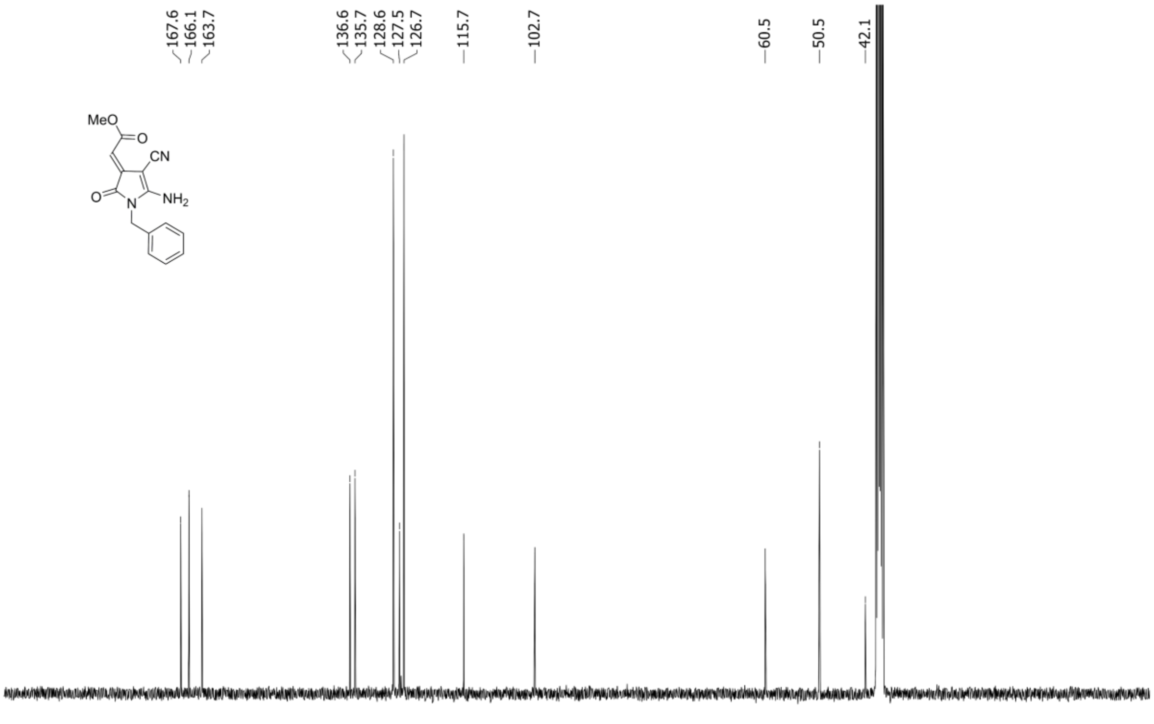

Figure S20. ^{1}H-^{13}C HSQC spectrum (DMSO-d_6) of **5a**

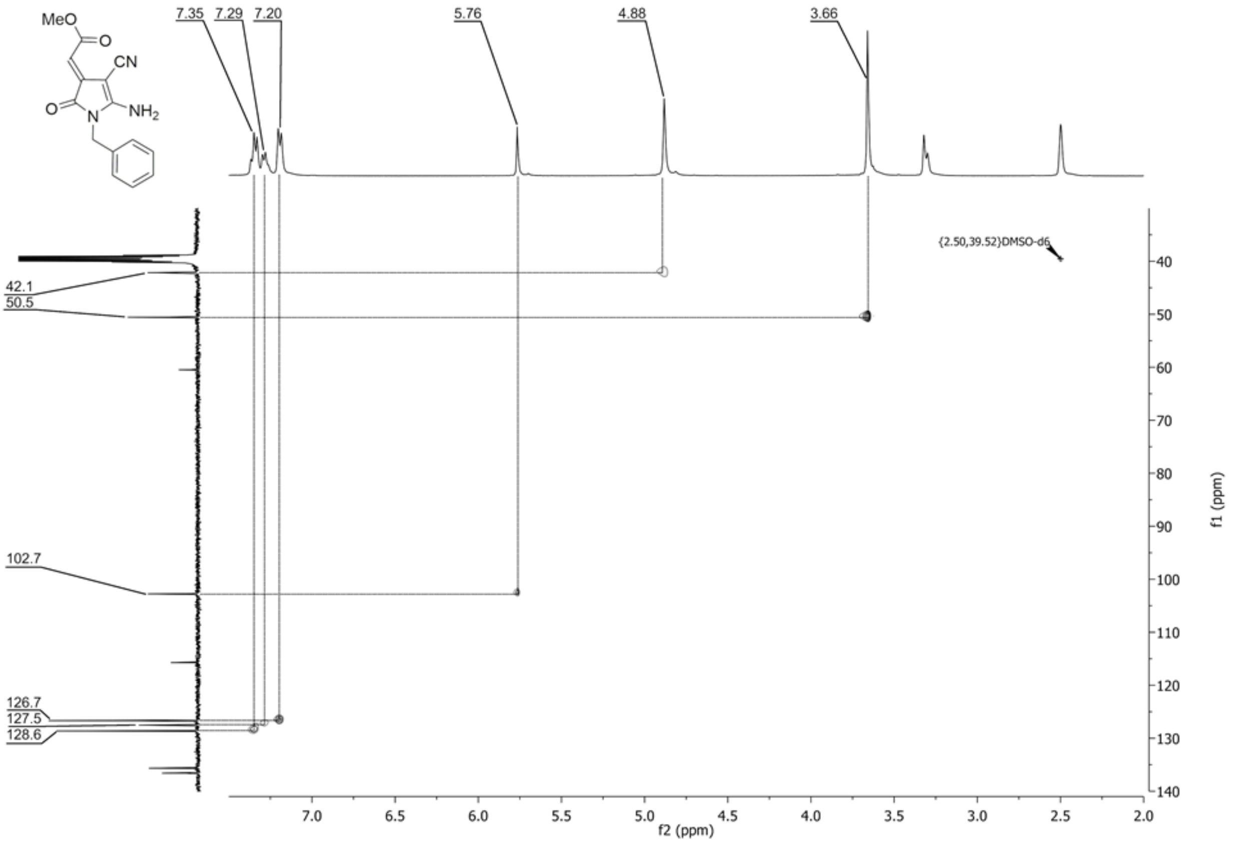

Figure S21. ^{1}H-^{13}C HMBC spectrum (DMSO-d_6) of **5a**

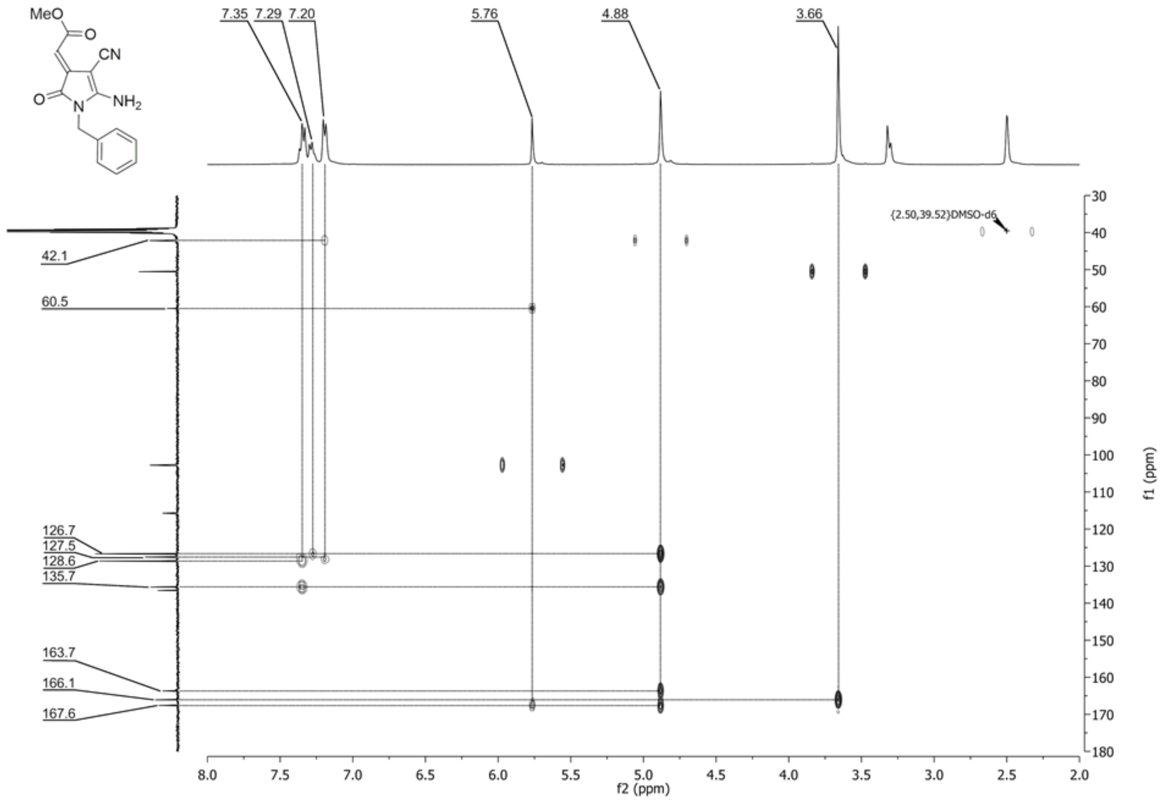

Figure S22. ^{1}H NMR spectrum (DMSO-d_6) of **5b**

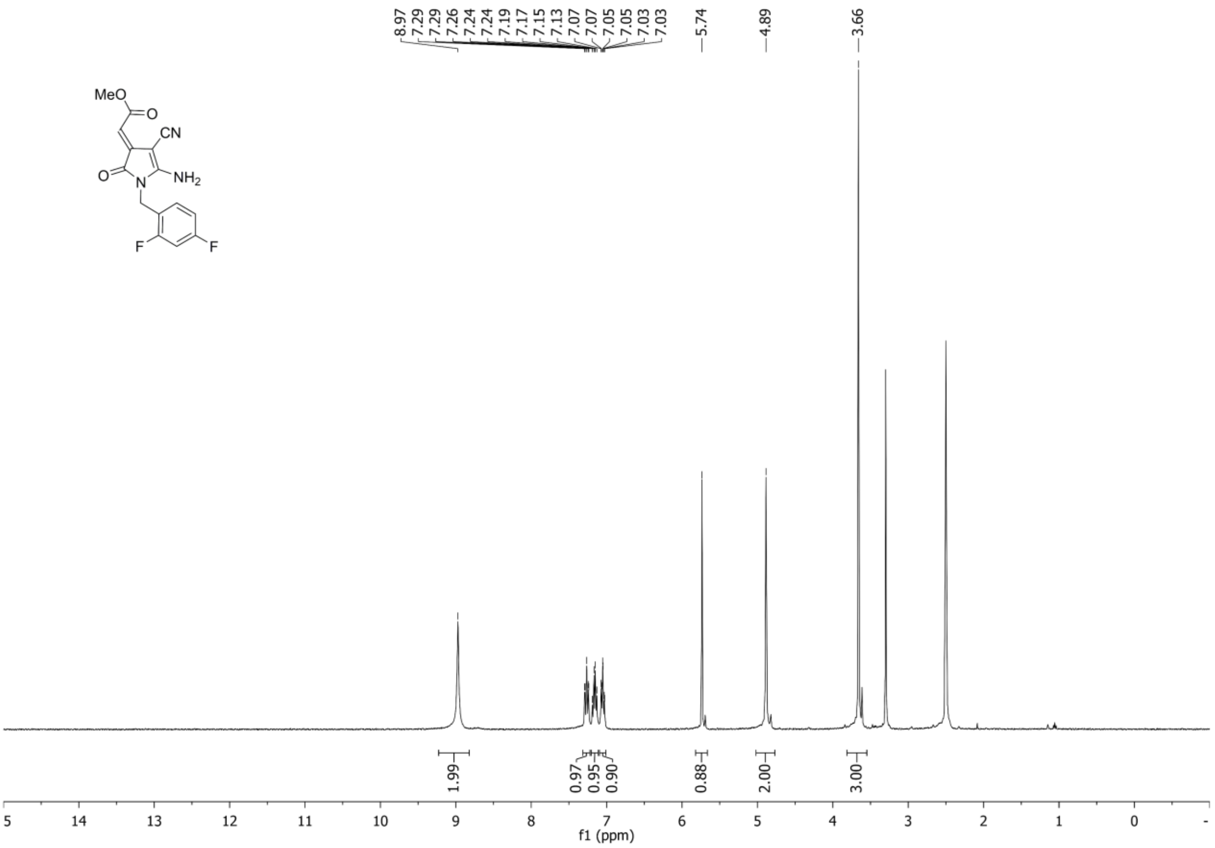

Figure S23. ^{13}C NMR spectrum (DMSO-d_6) of **5b**

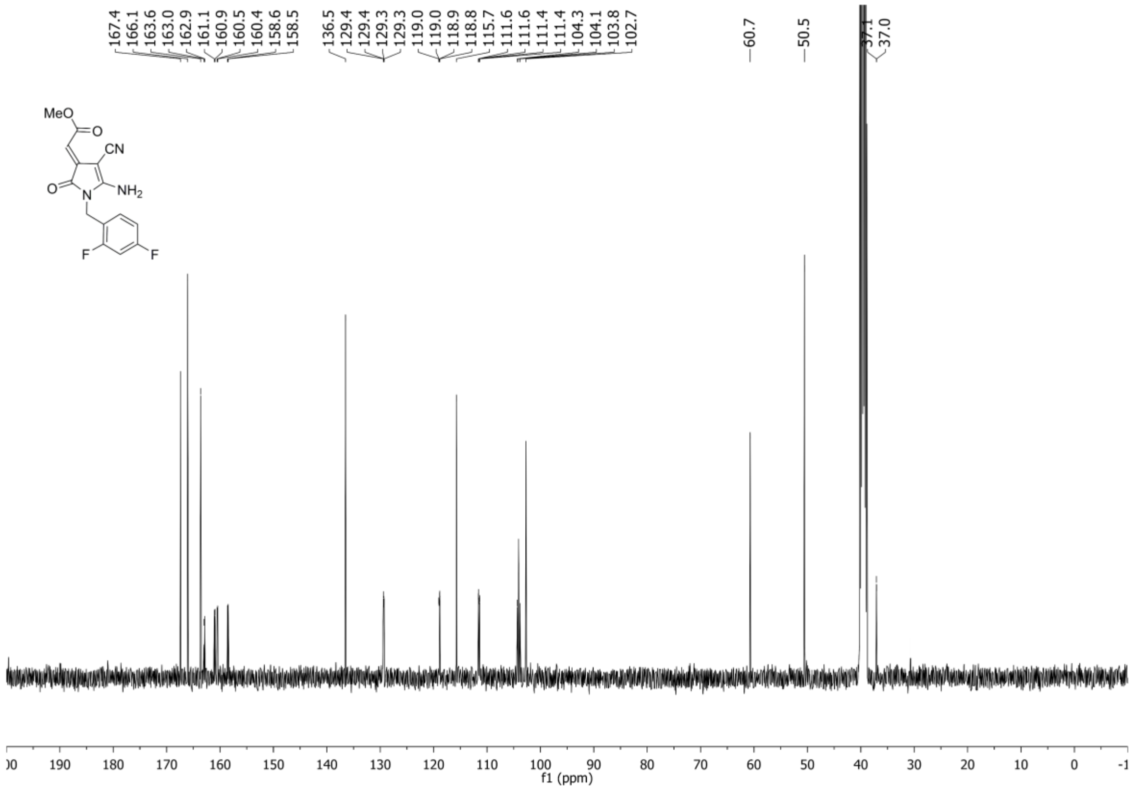

Figure S24. ^{1}H NMR spectrum (DMSO-d_6) of **5c**

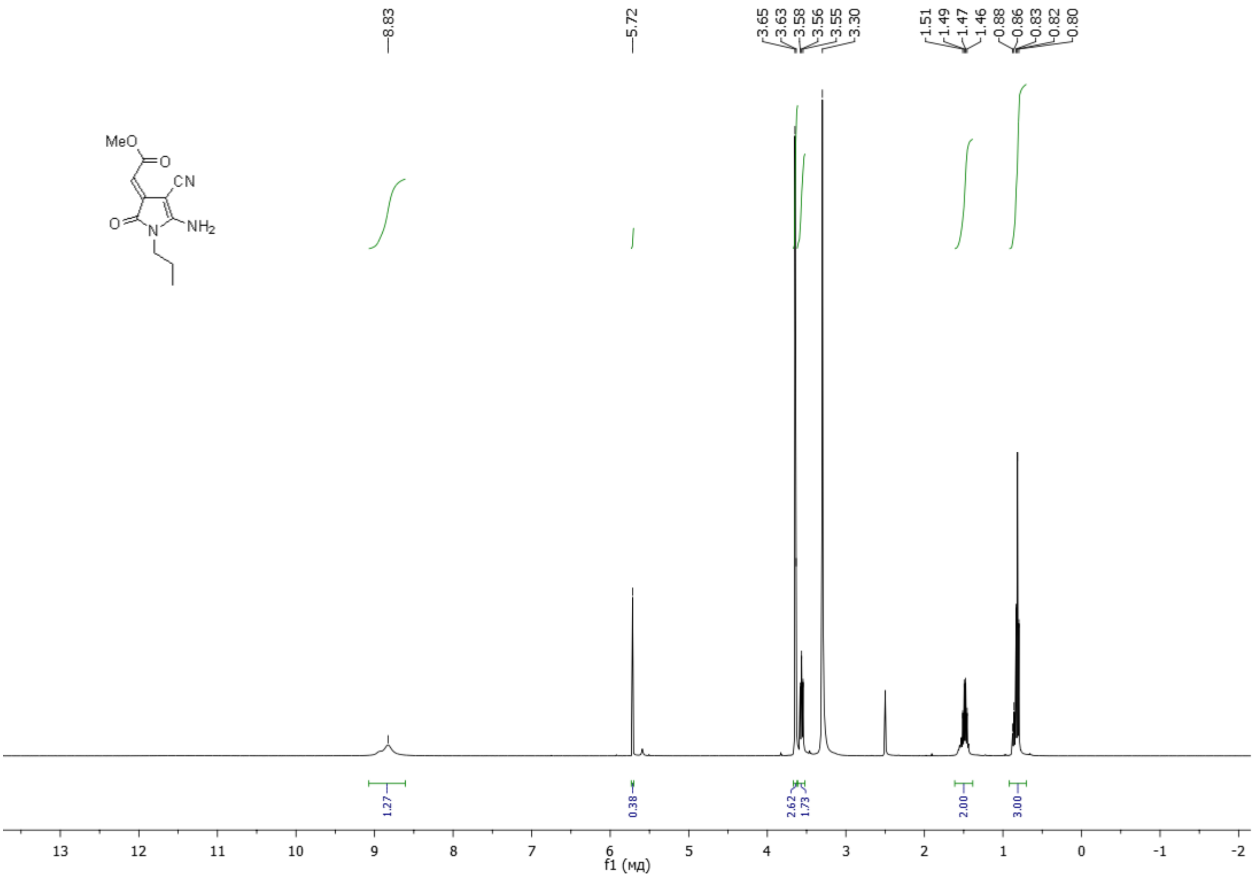

Figure S25. ^{13}C NMR spectrum (DMSO-d_6) of **5c**

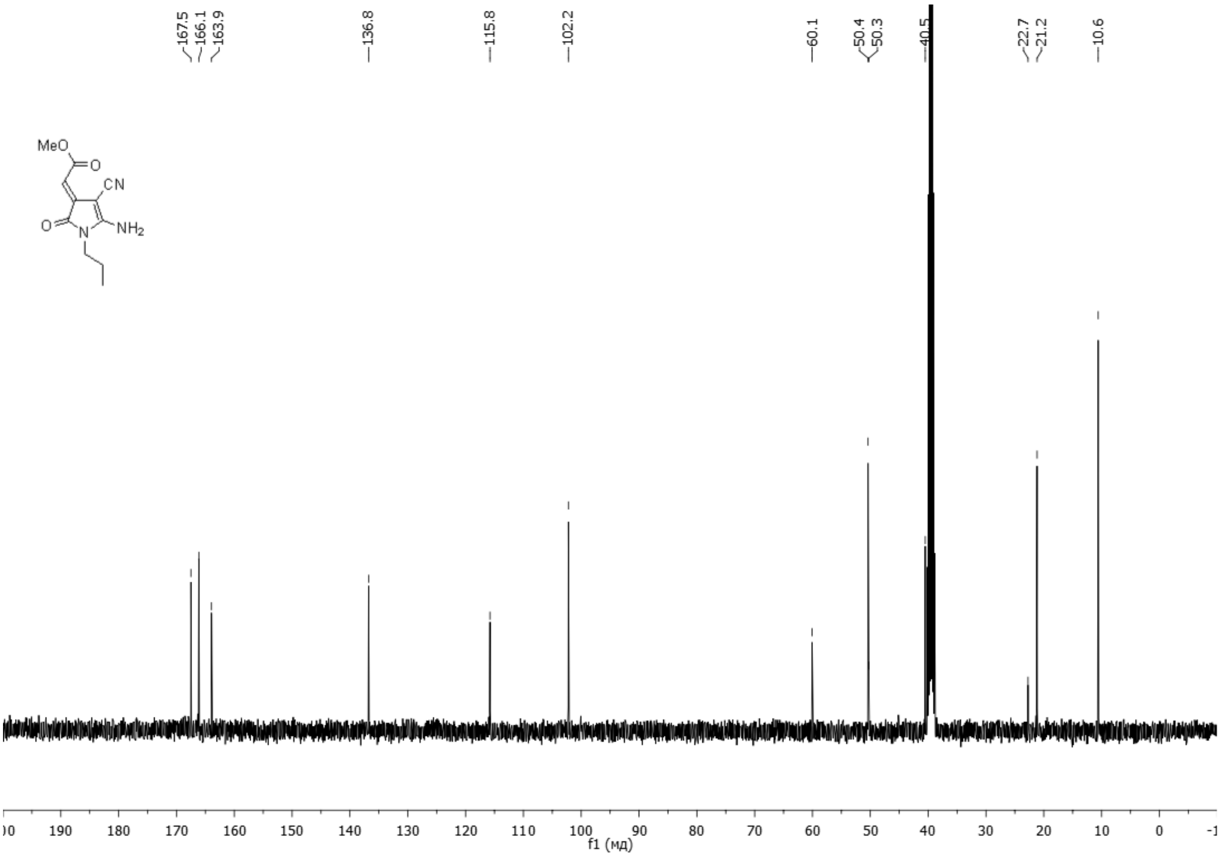

Figure S26. ^{1}H NMR spectrum (DMSO-d_6) of **5d**

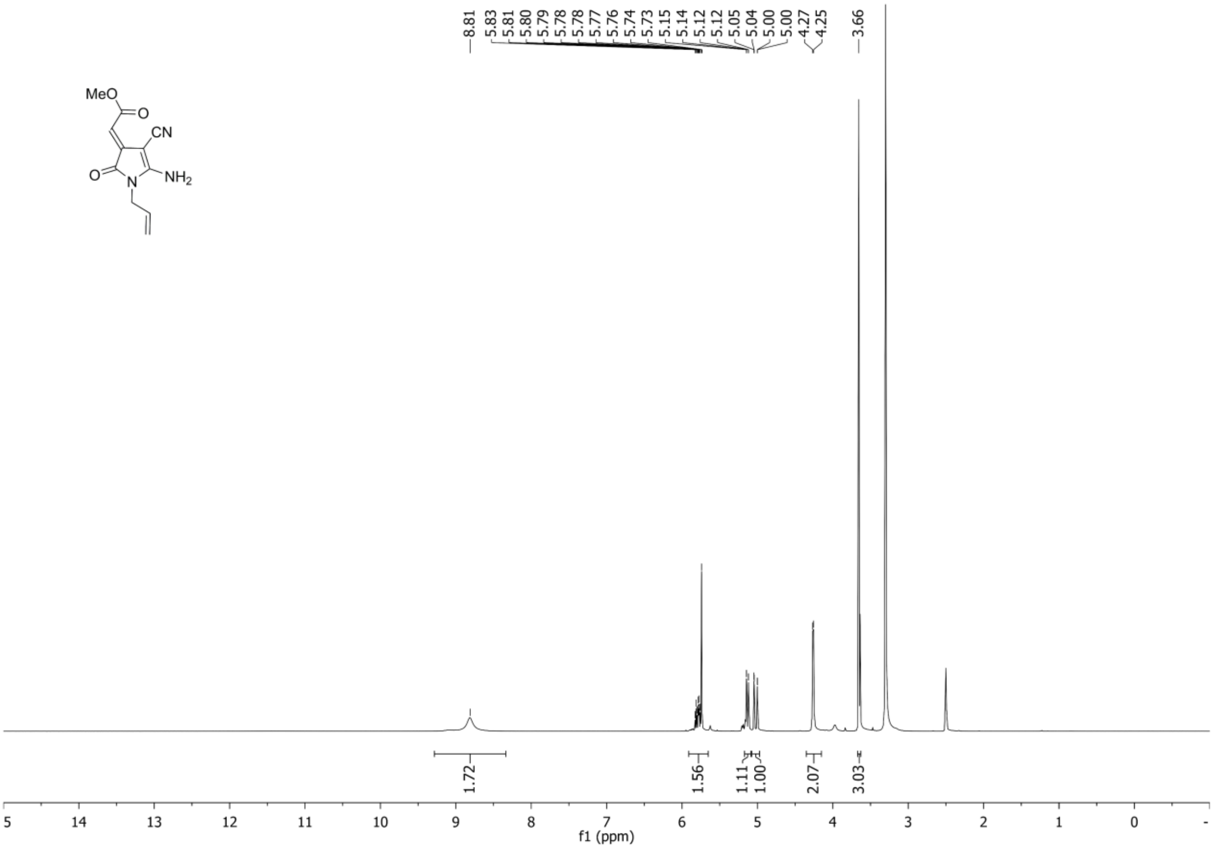

Figure S27. ^{13}C NMR spectrum (DMSO-d_6) of **5d**

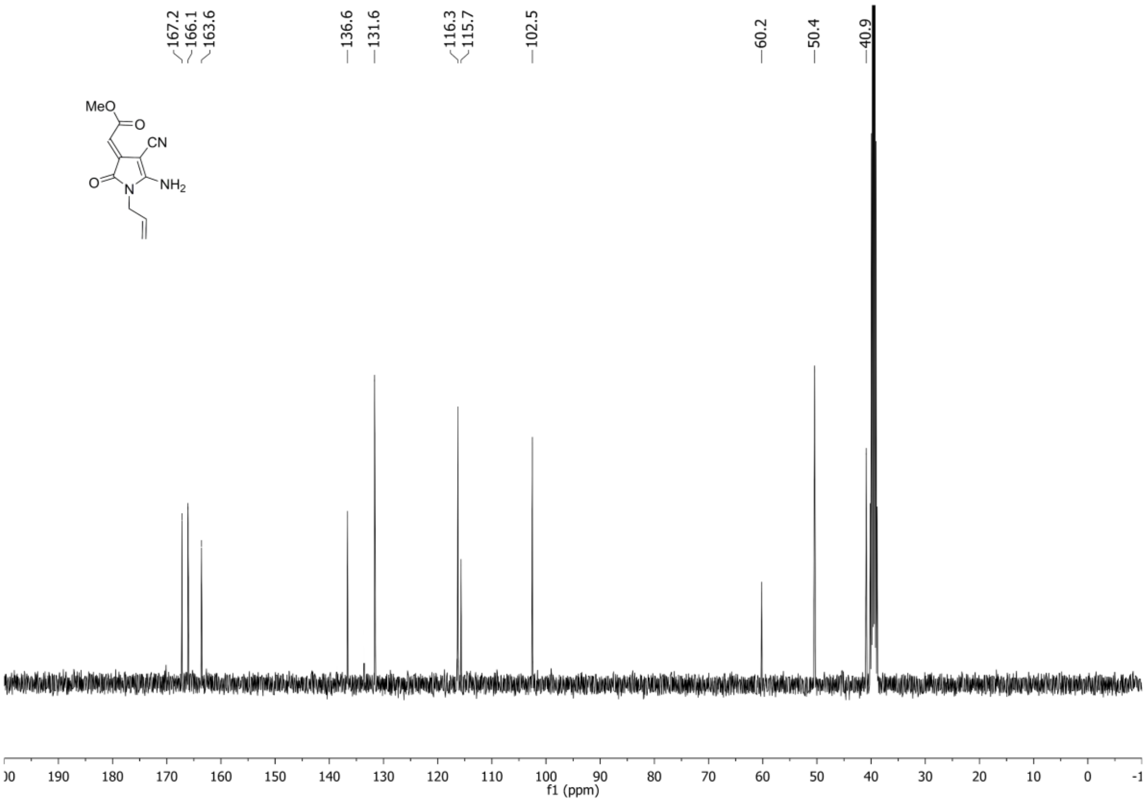

Figure S28. ^{1}H NMR spectrum (DMSO-d_6) of **5e**

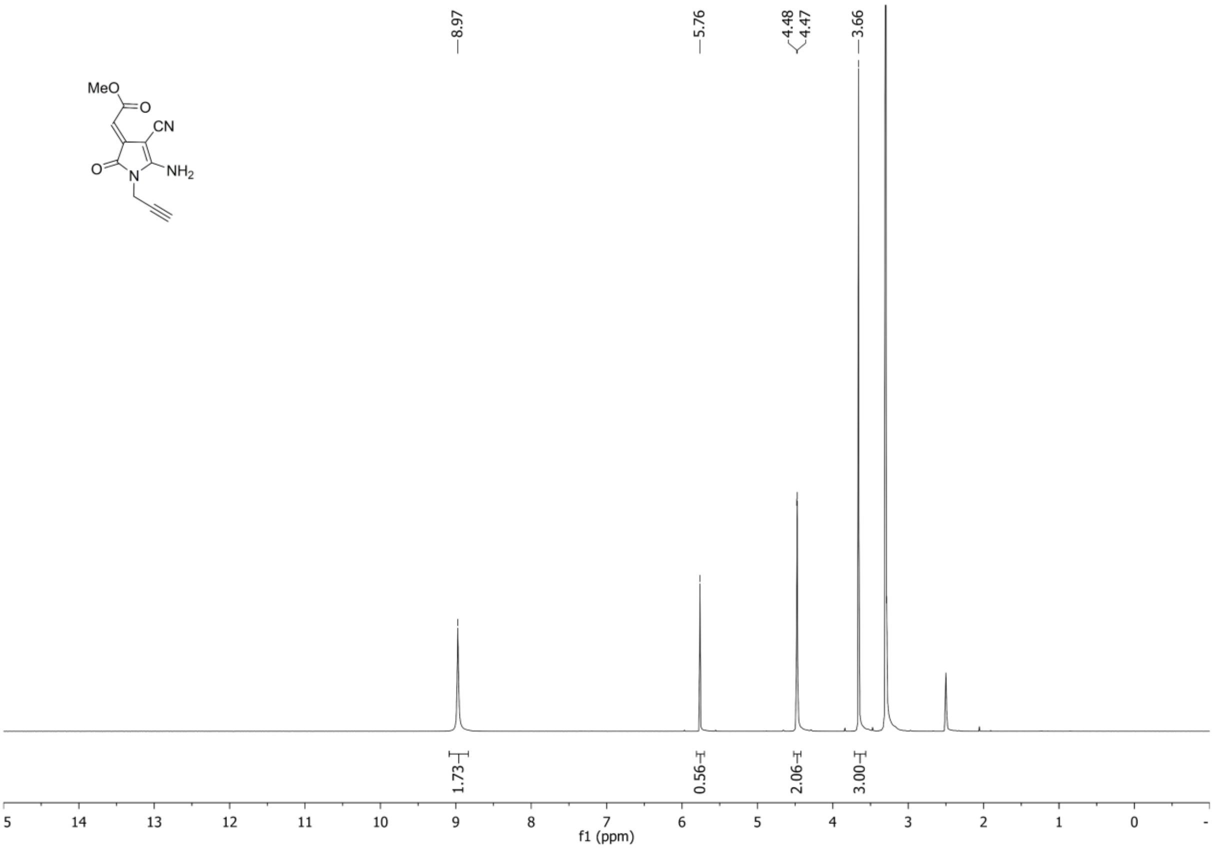

Figure S29. ^{13}C NMR spectrum (DMSO-d_6) of **5e**

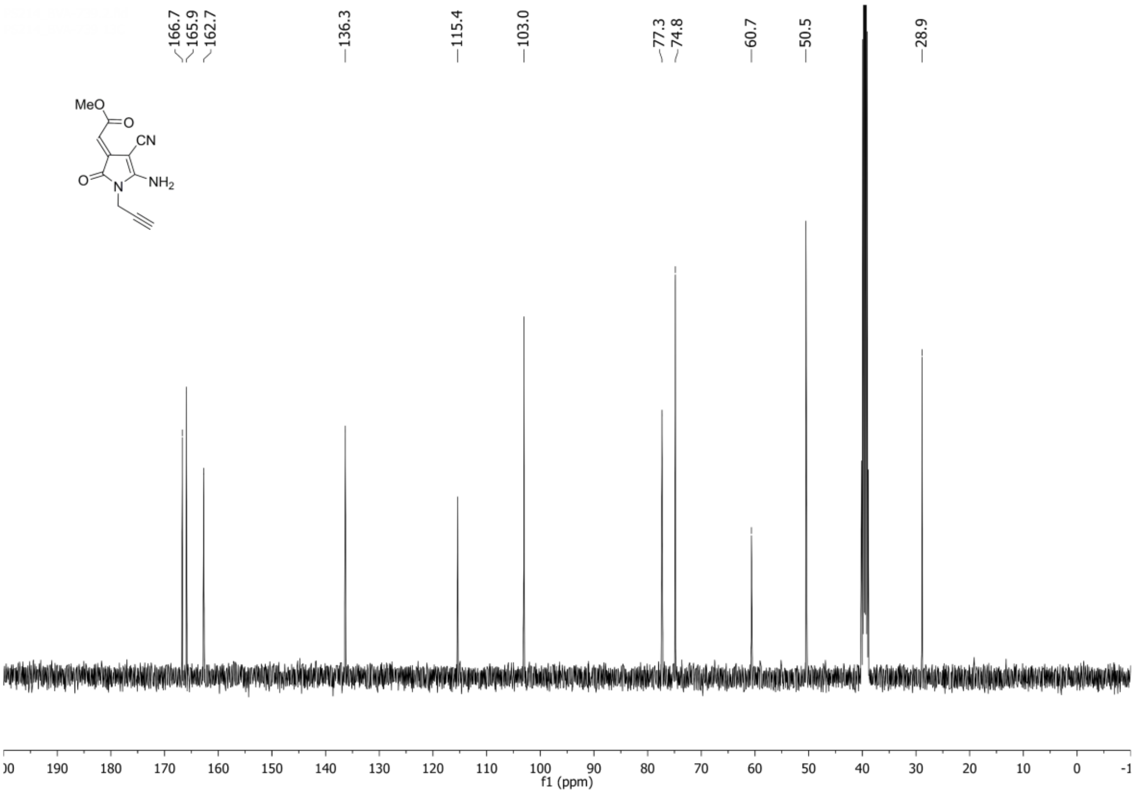

Figure S30. ^{1}H NMR spectrum (DMSO-d_6) of **5f**

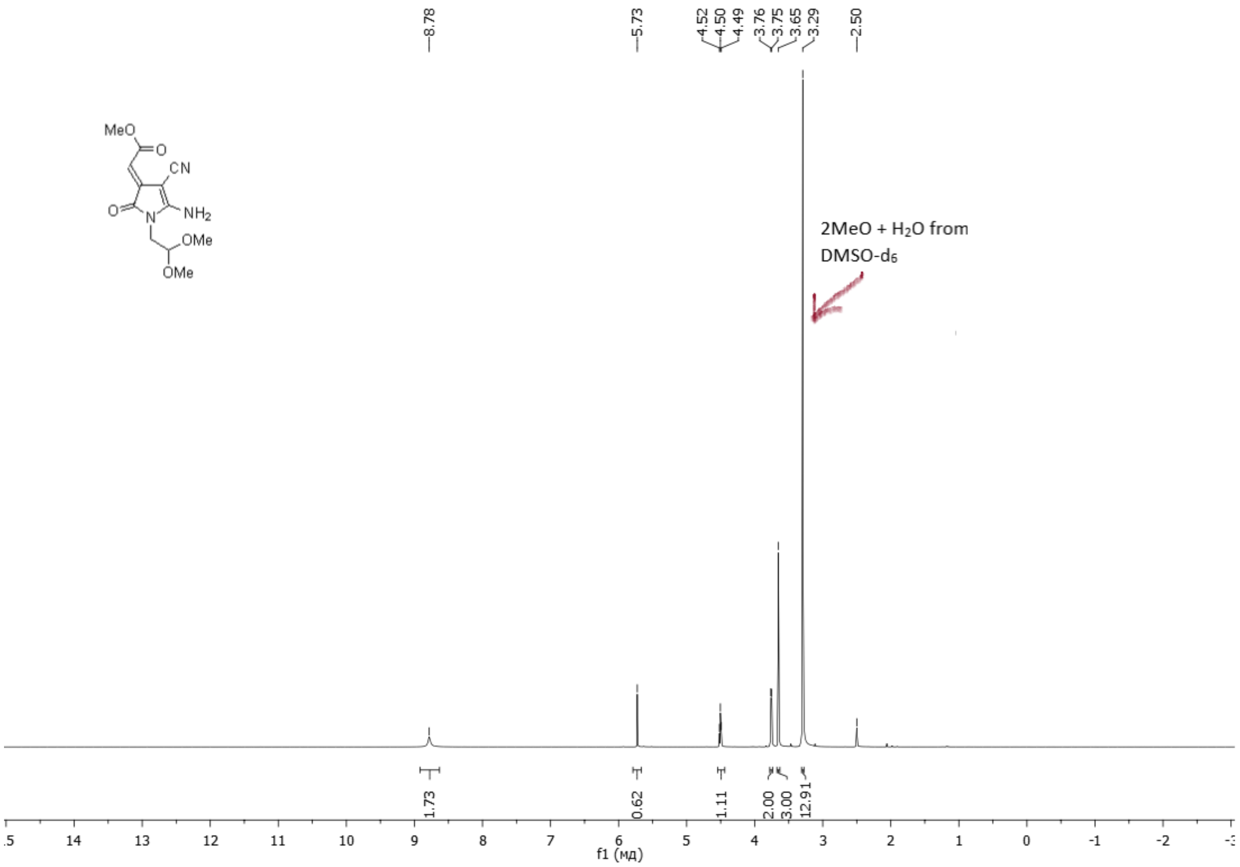

Figure S31. ^{13}C NMR spectrum (DMSO-d_6) of **5f**

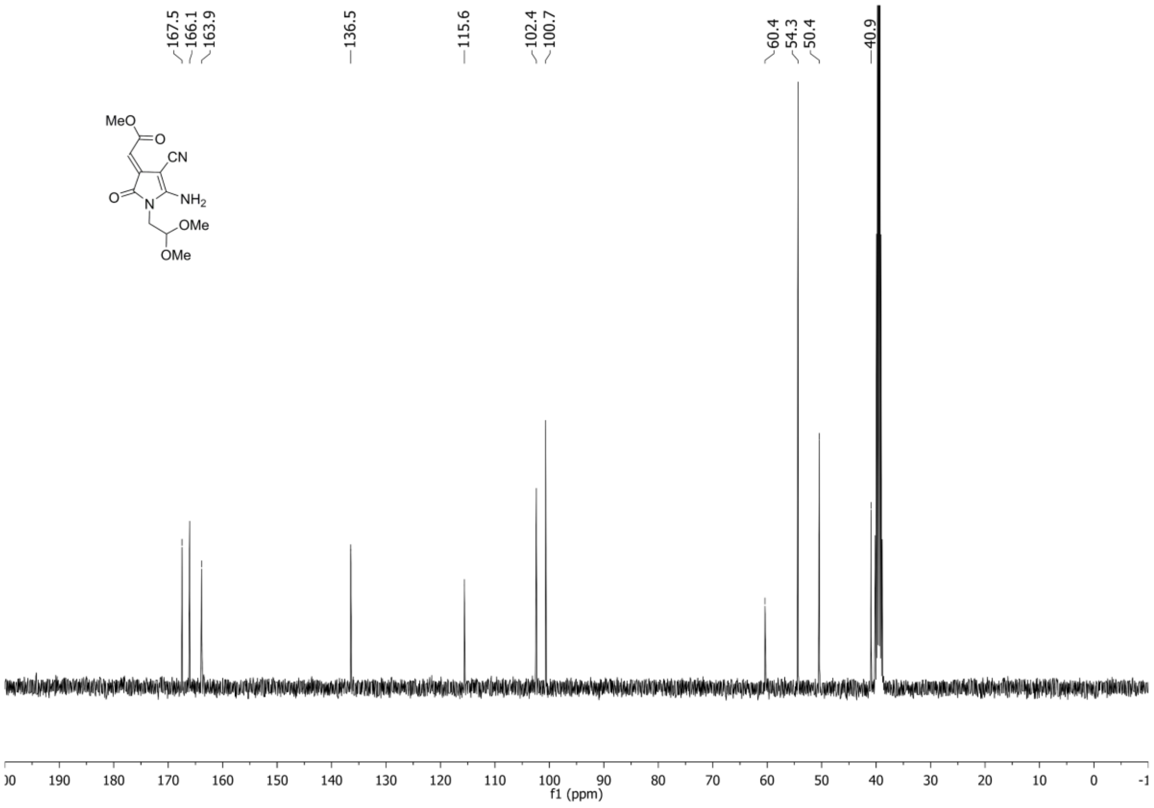

Figure S32. ^{1}H NMR spectrum ($CDCl_3$) of **6a**

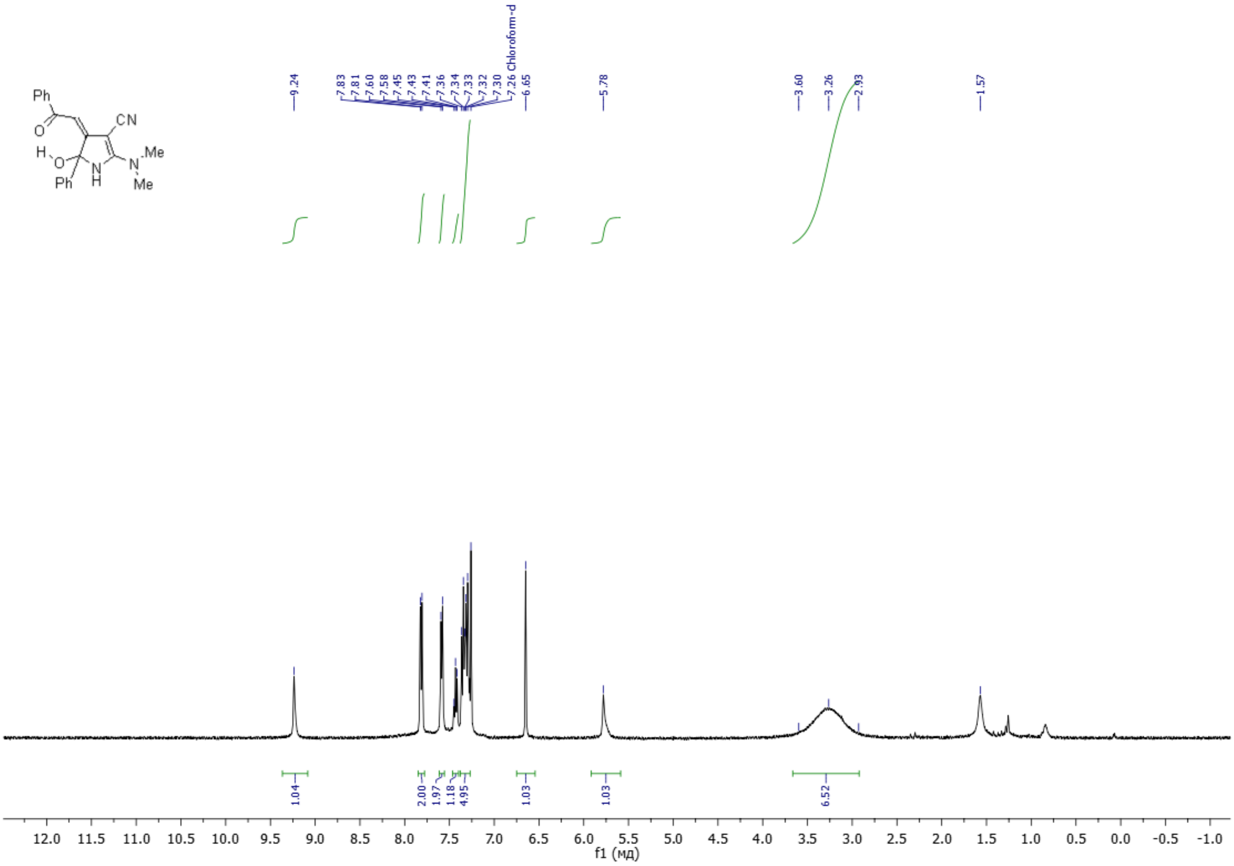

Figure S33. ^{13}C NMR spectrum ($CDCl_3$) of **6a**

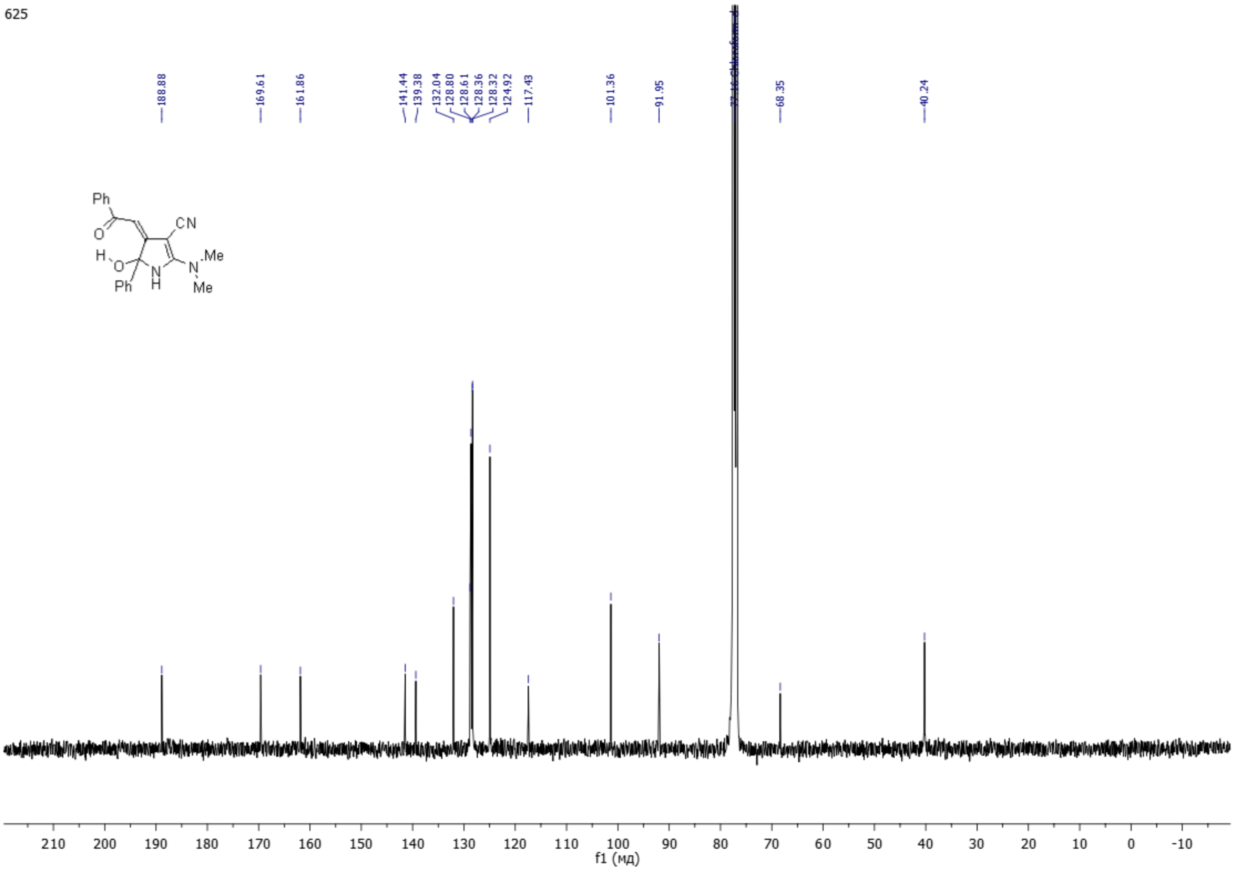

Figure S34. ^{1}H NMR spectrum ($CDCl_3$) of **6b**

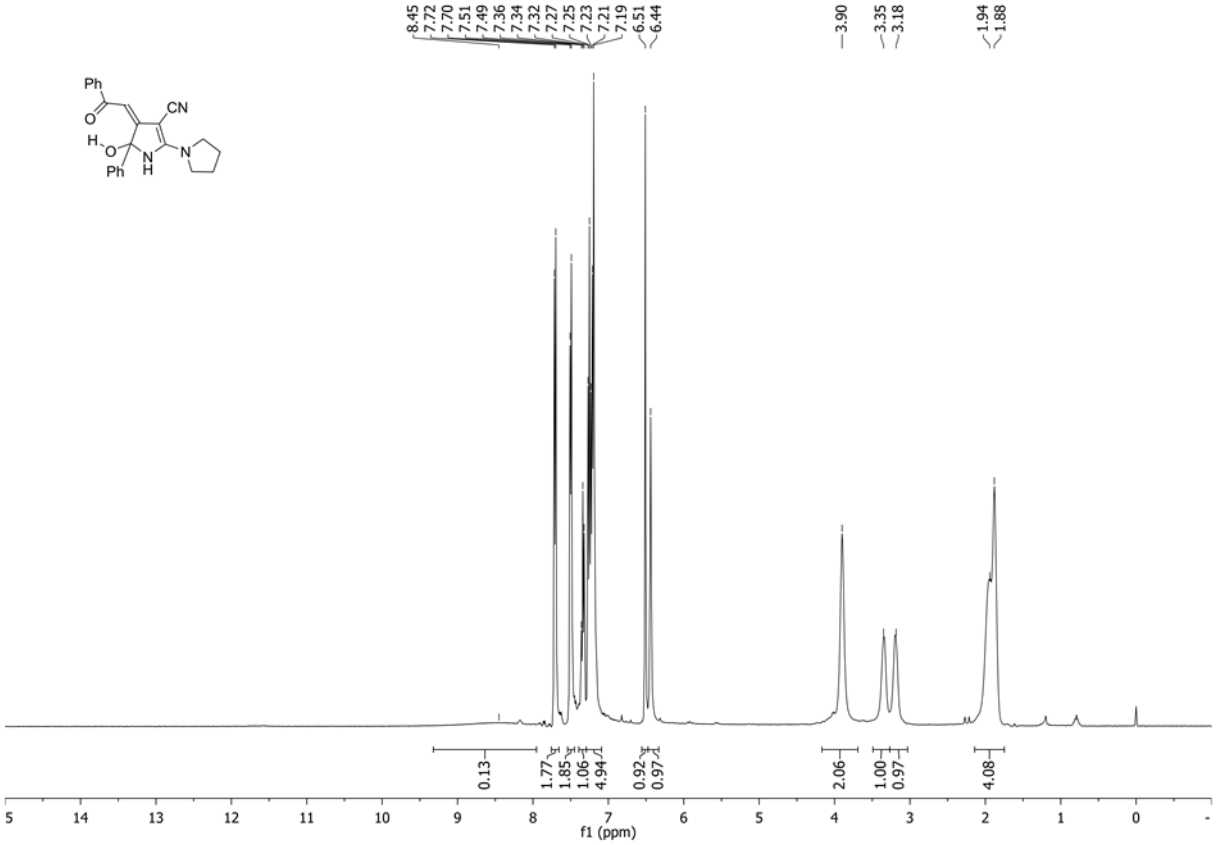

Figure S35. ^{13}C NMR spectrum ($CDCl_3$) of **6b**

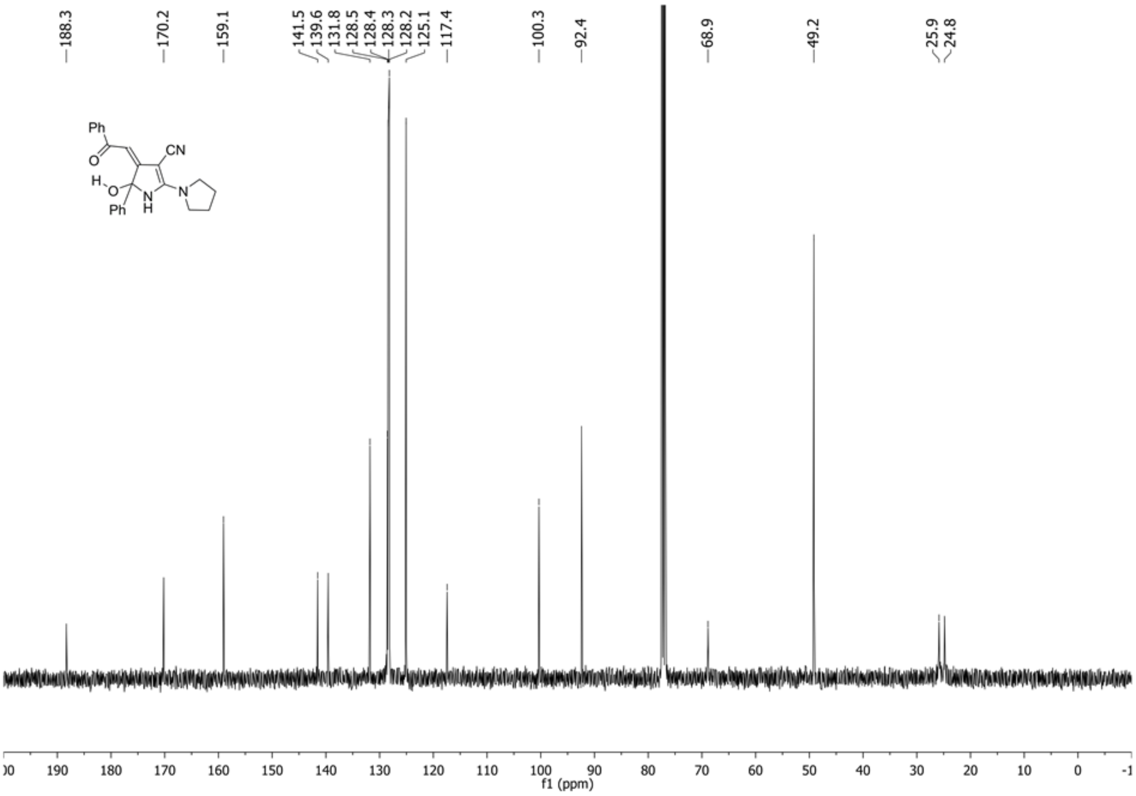

Figure S36. ^{1}H-^{13}C HSQC spectrum of **6b**

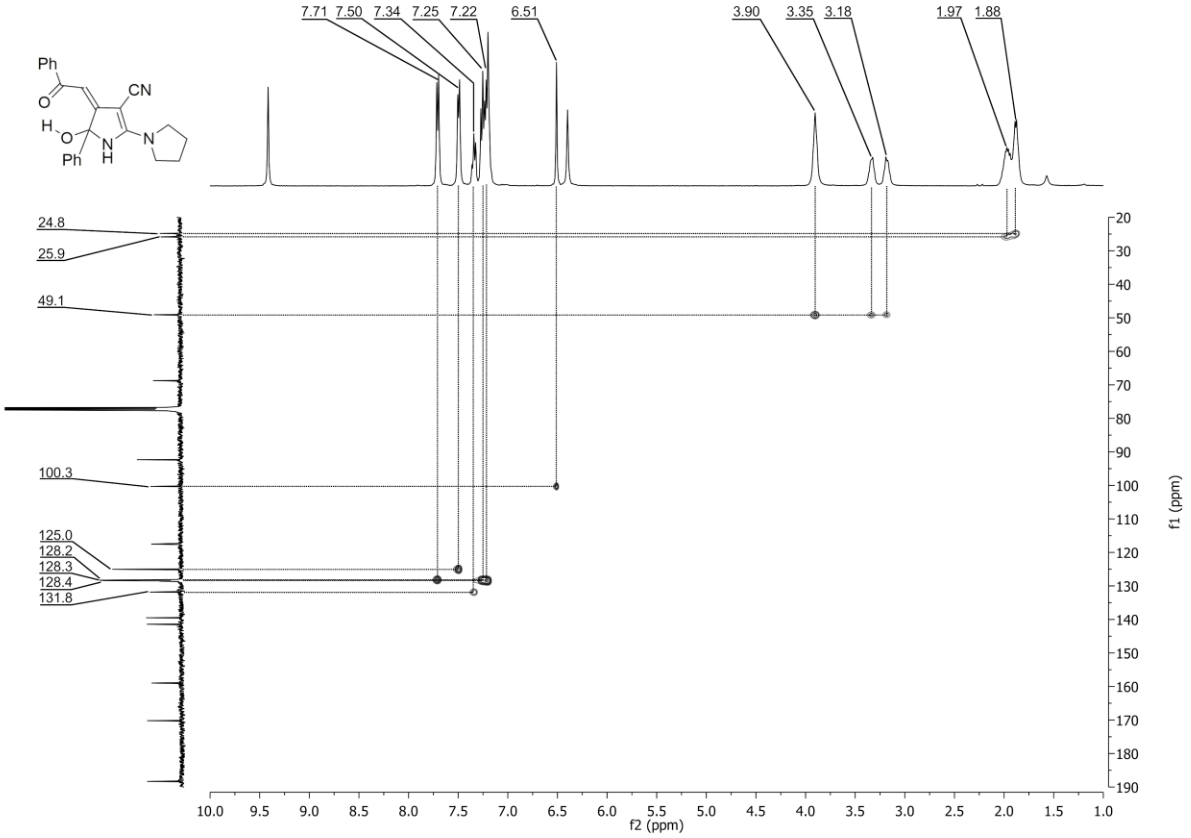

Figure S37. ^{1}H-^{13}C HMBC spectrum of **6b**

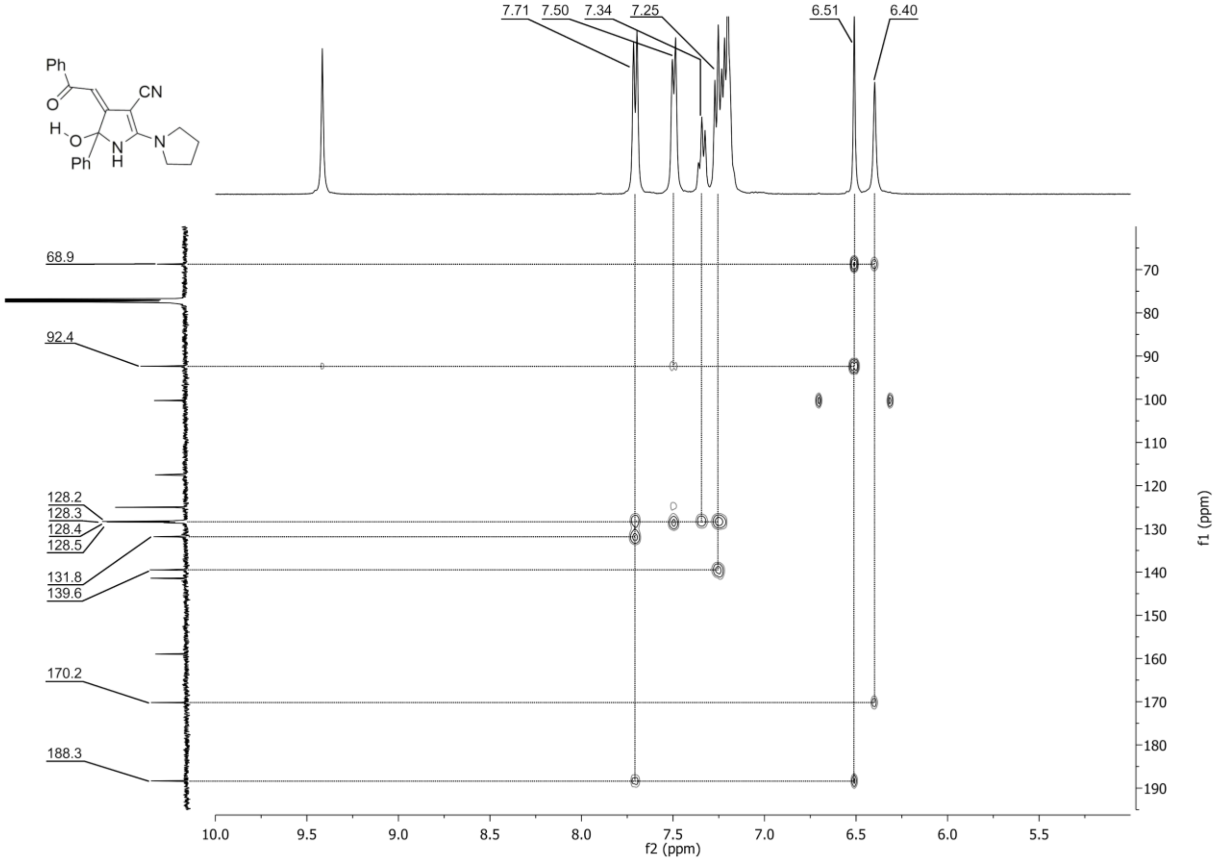

Figure S38. ^{1}H NMR spectrum ($CDCl_3$) of **6c**

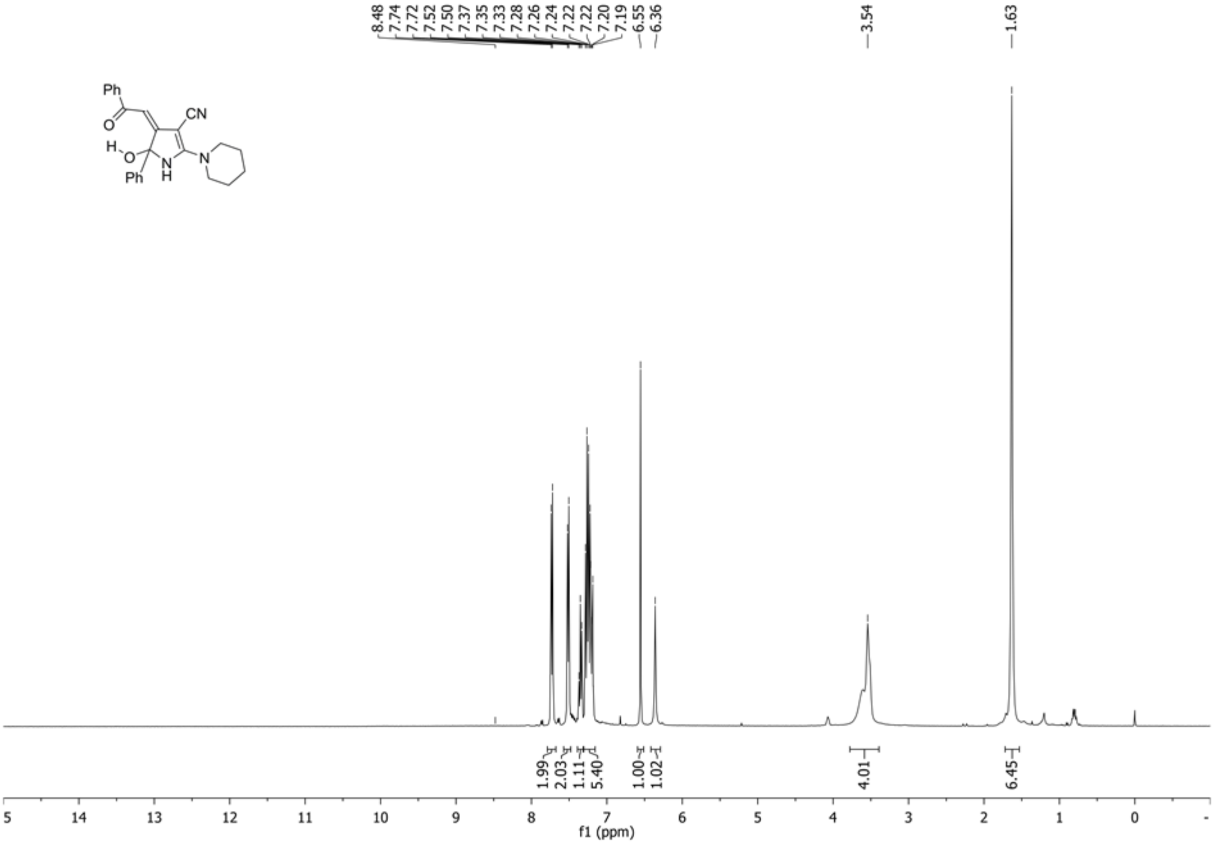

Figure S39. ^{13}C NMR spectrum ($CDCl_3$) of **6c**

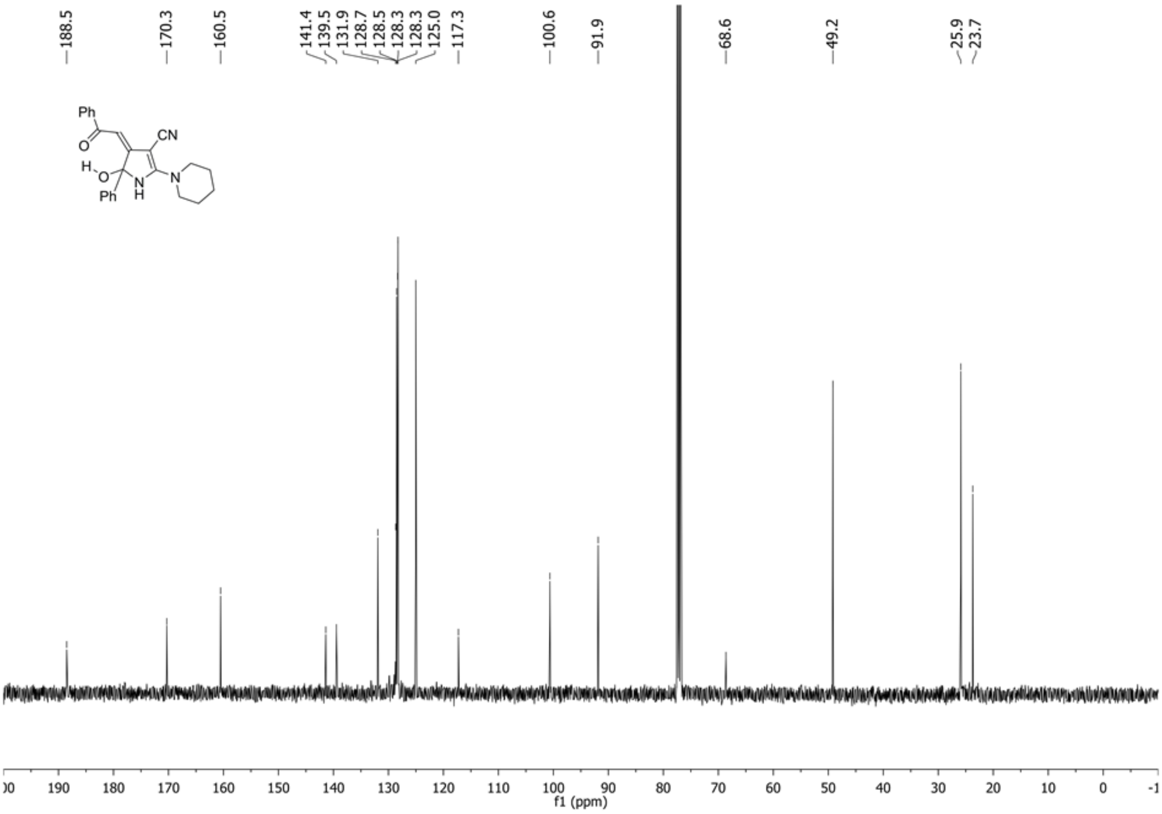

Figure S40. ^{1}H NMR spectrum ($CDCl_3$) of **6d**

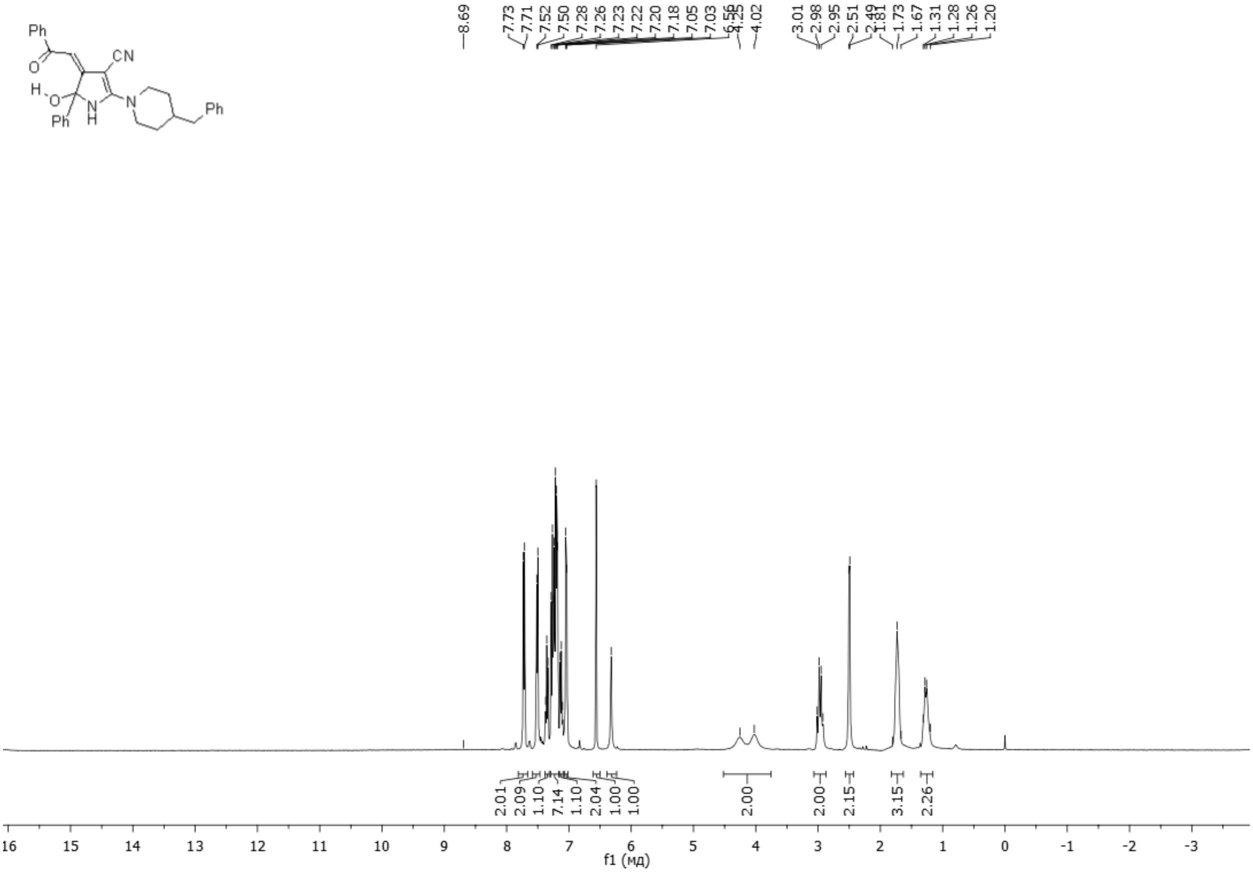

Figure S41. ^{13}C NMR spectrum ($CDCl_3$) of **6d**

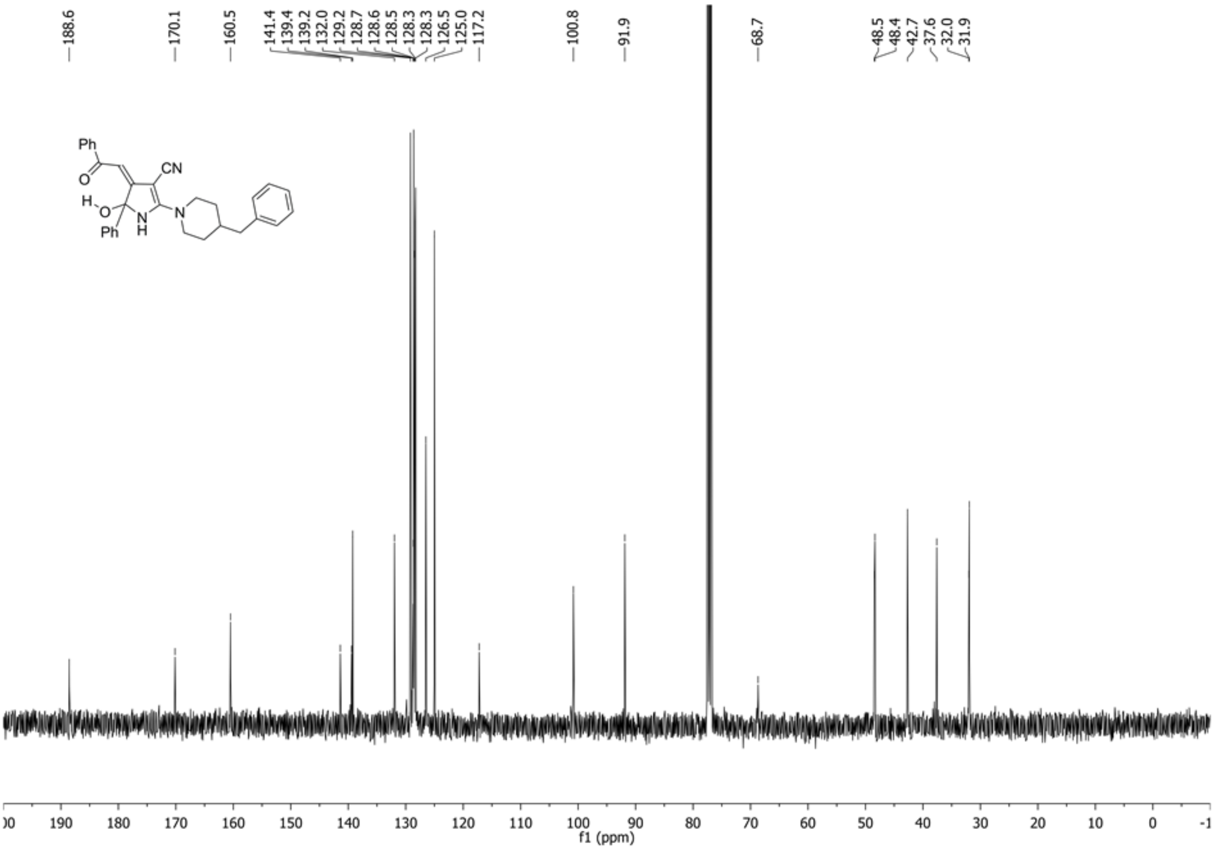

Figure S42 ^{1}H NMR spectrum ($CDCl_3$) of **6e**

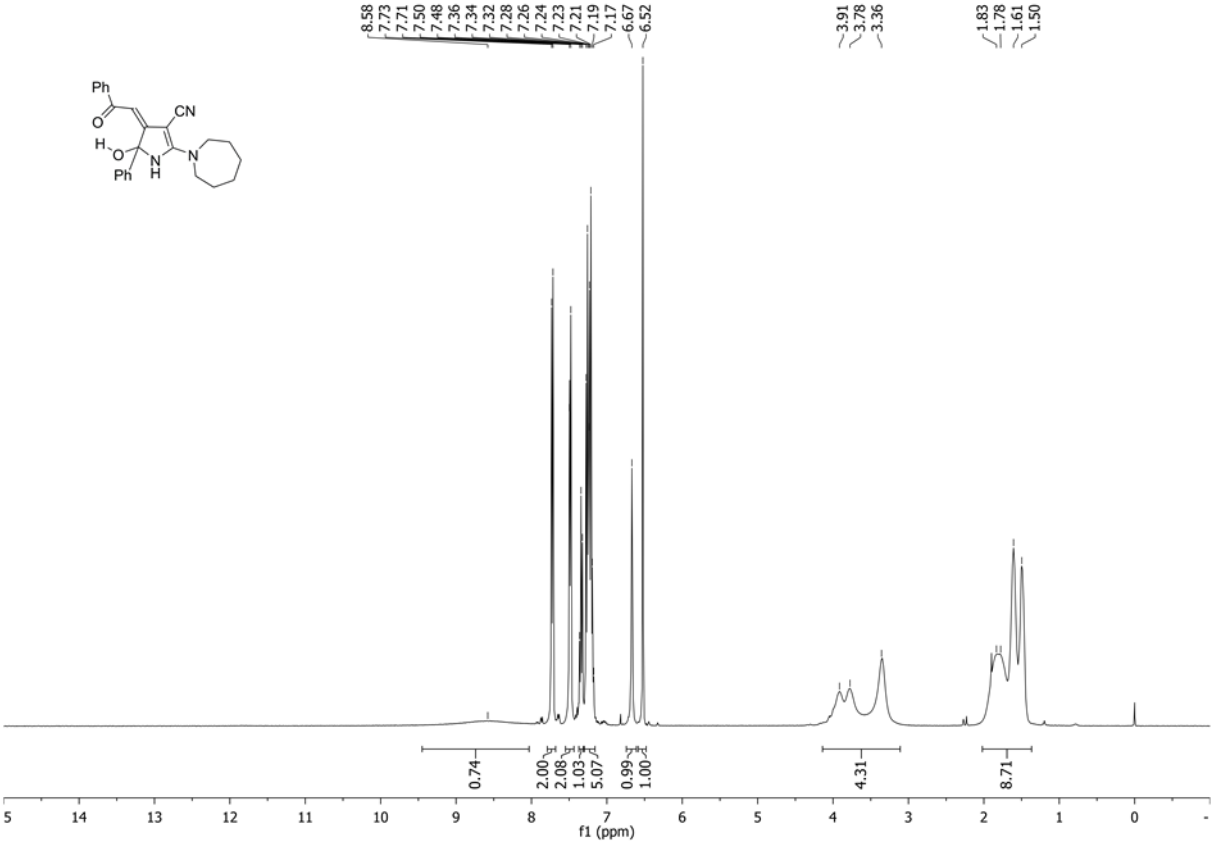

Figure S43 ^{13}C NMR spectrum ($CDCl_3$) of **6e**

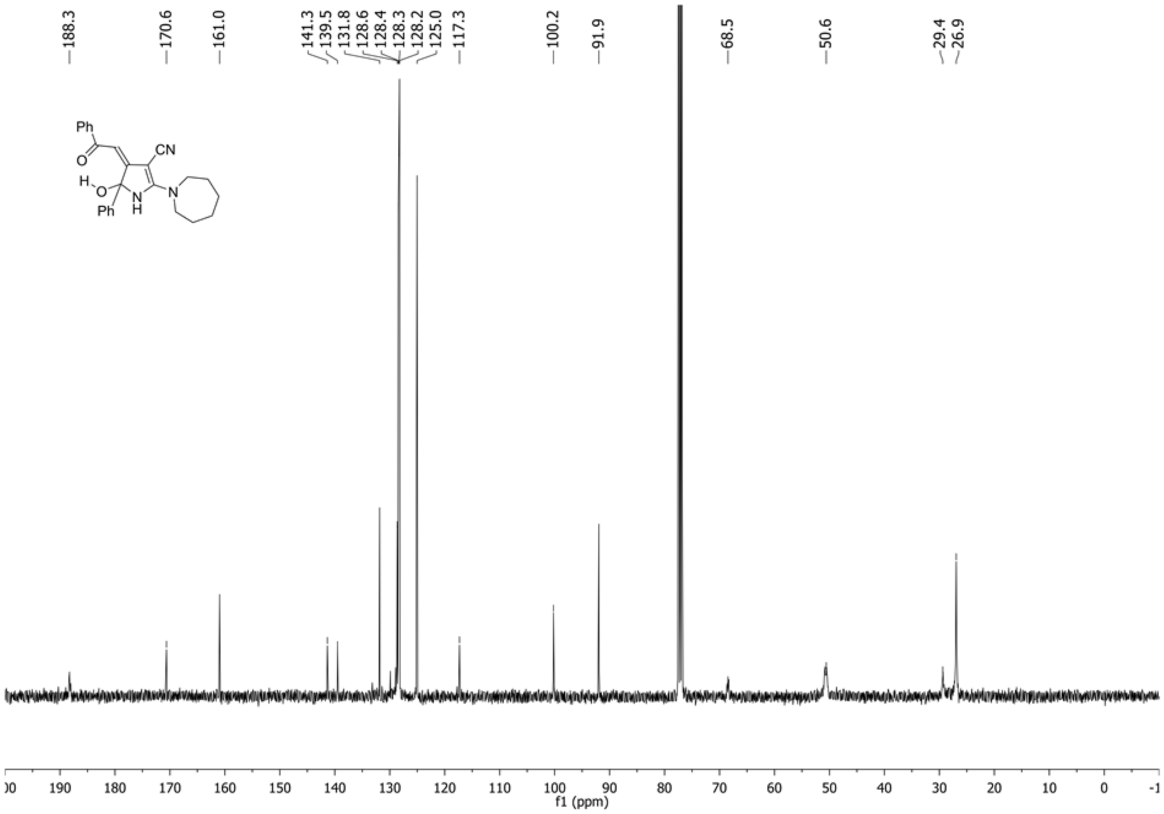

Figure S44 ^{1}H NMR spectrum ($CDCl_3$) of **6f**

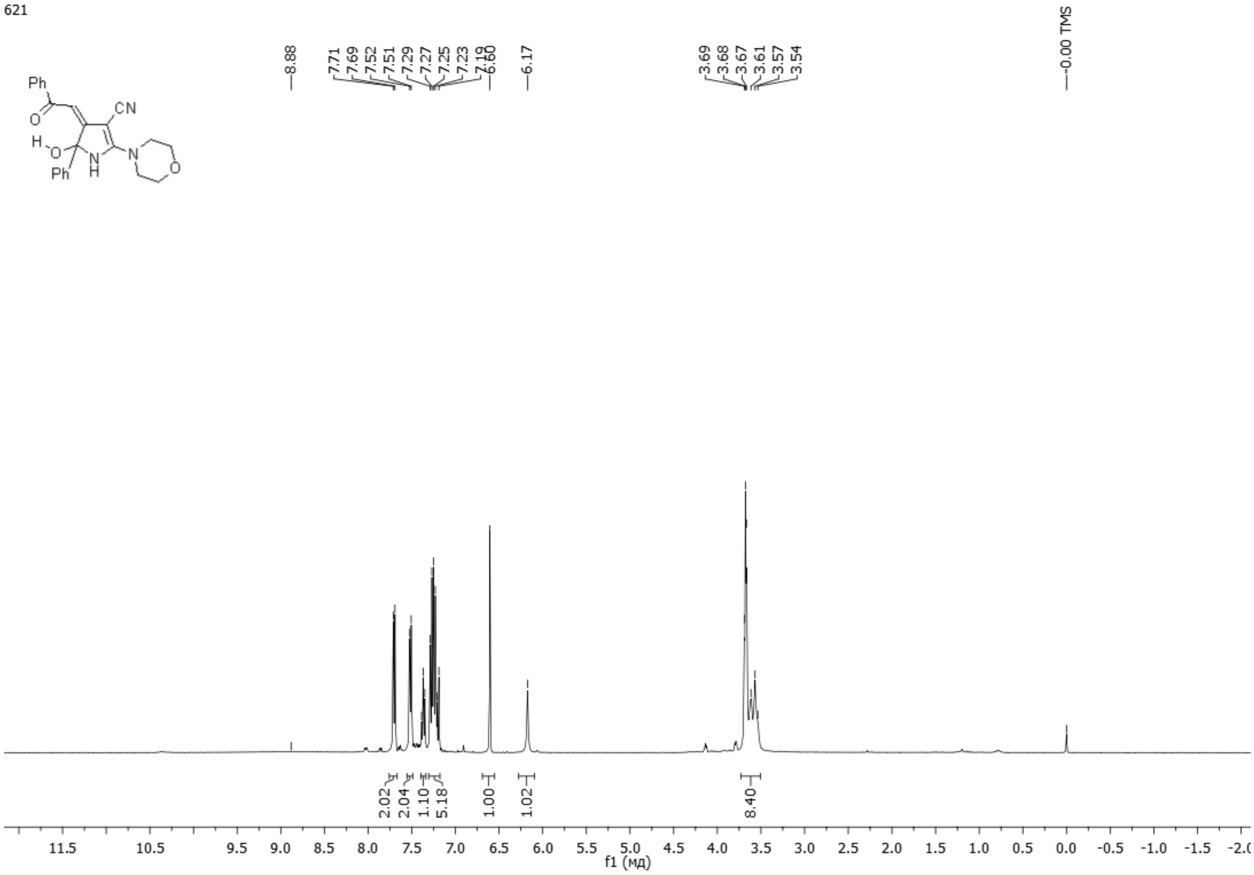

Figure S45 ^{13}C NMR spectrum ($CDCl_3$) of **6f**

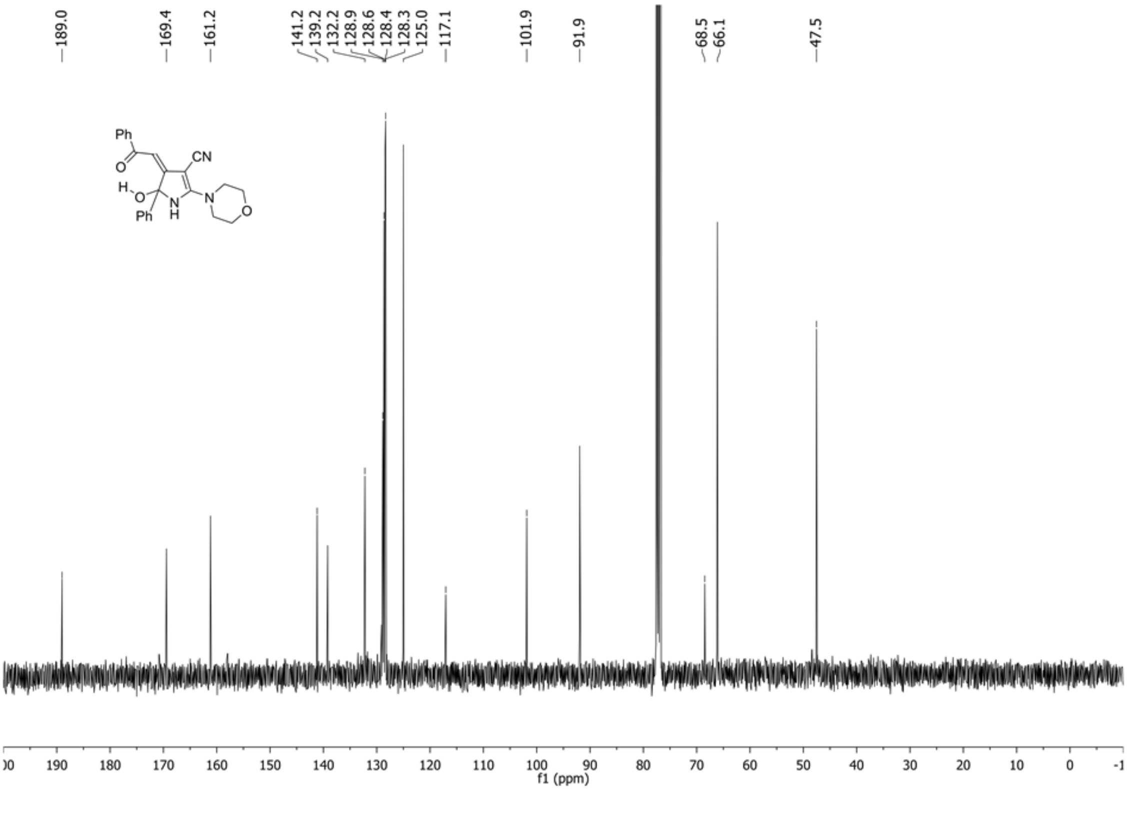

Figure S46 ^{1}H NMR spectrum ($CDCl_3$) of **6g**

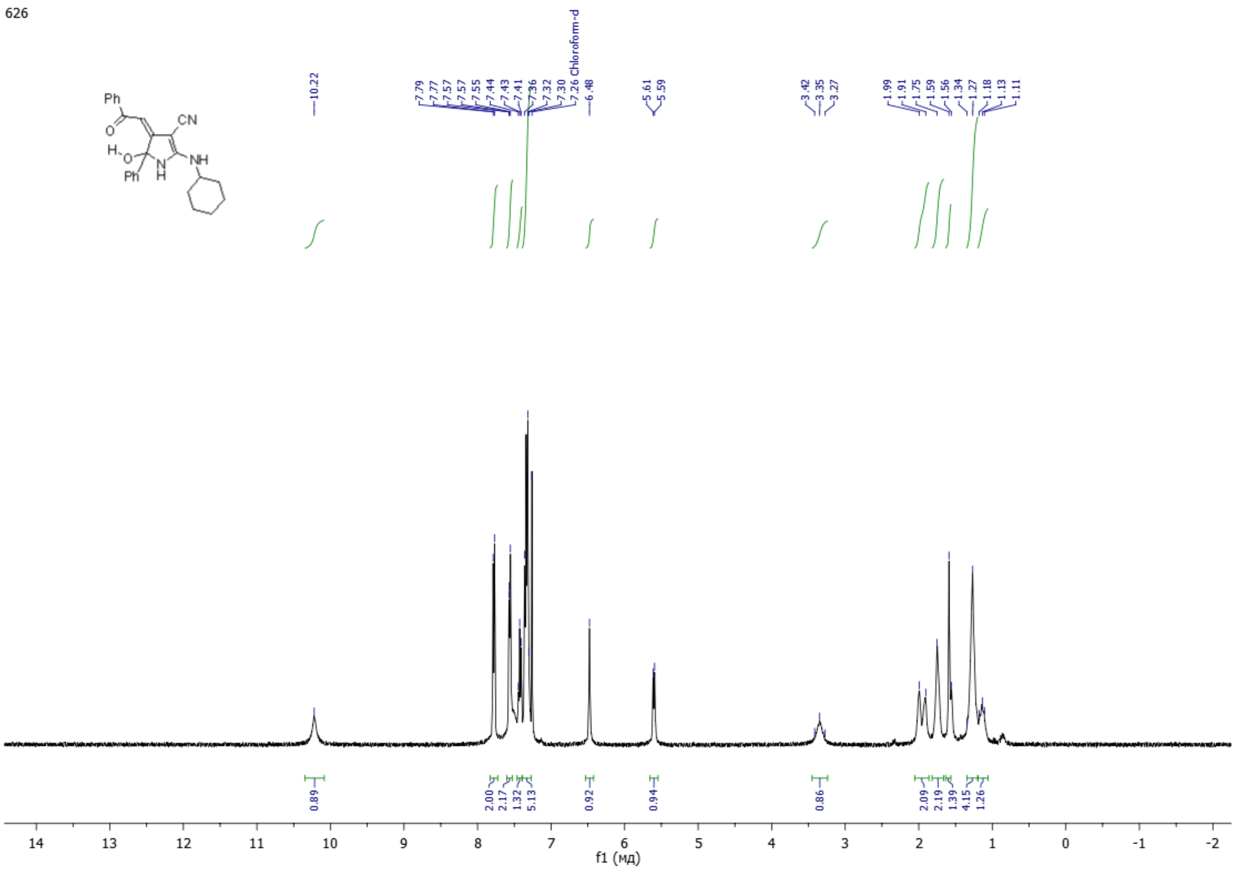

Figure S47 ^{13}C NMR spectrum ($CDCl_3$) of **6g**

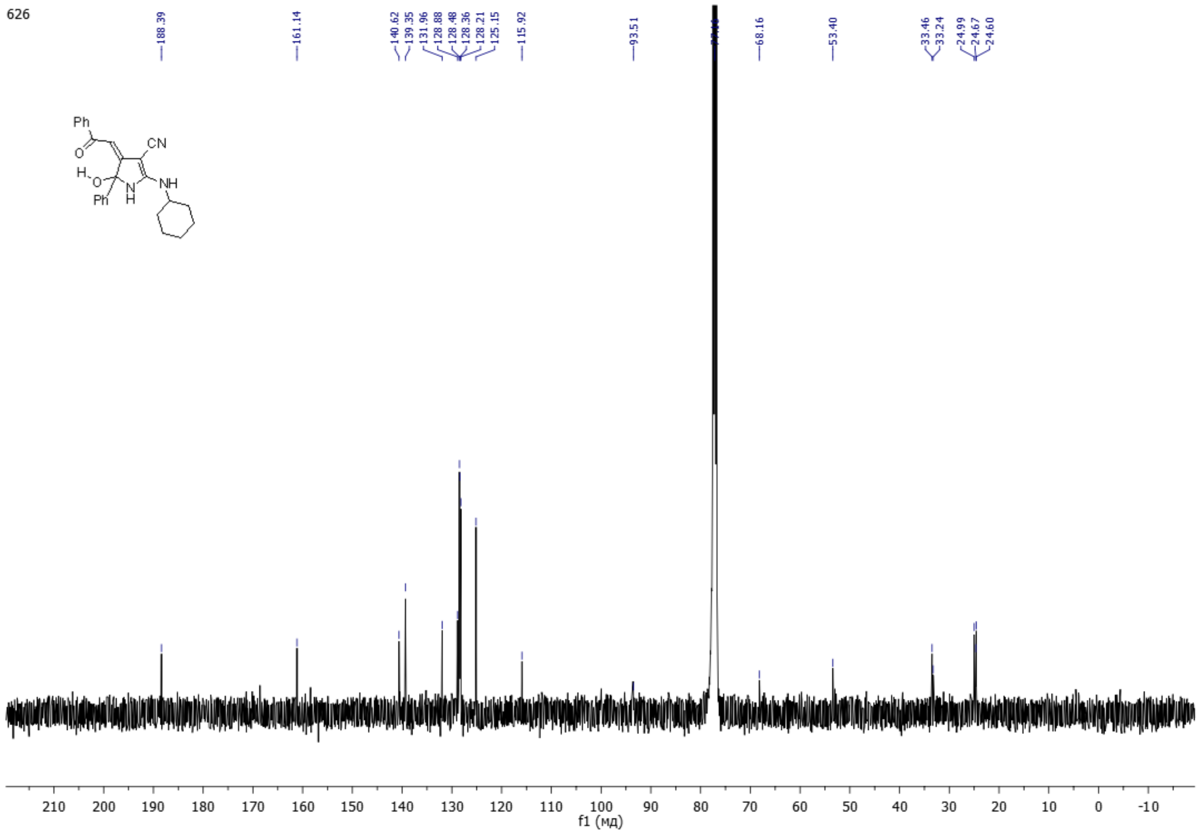

Figure S48 ^{1}H NMR spectrum ($CDCl_3$) of **7a**

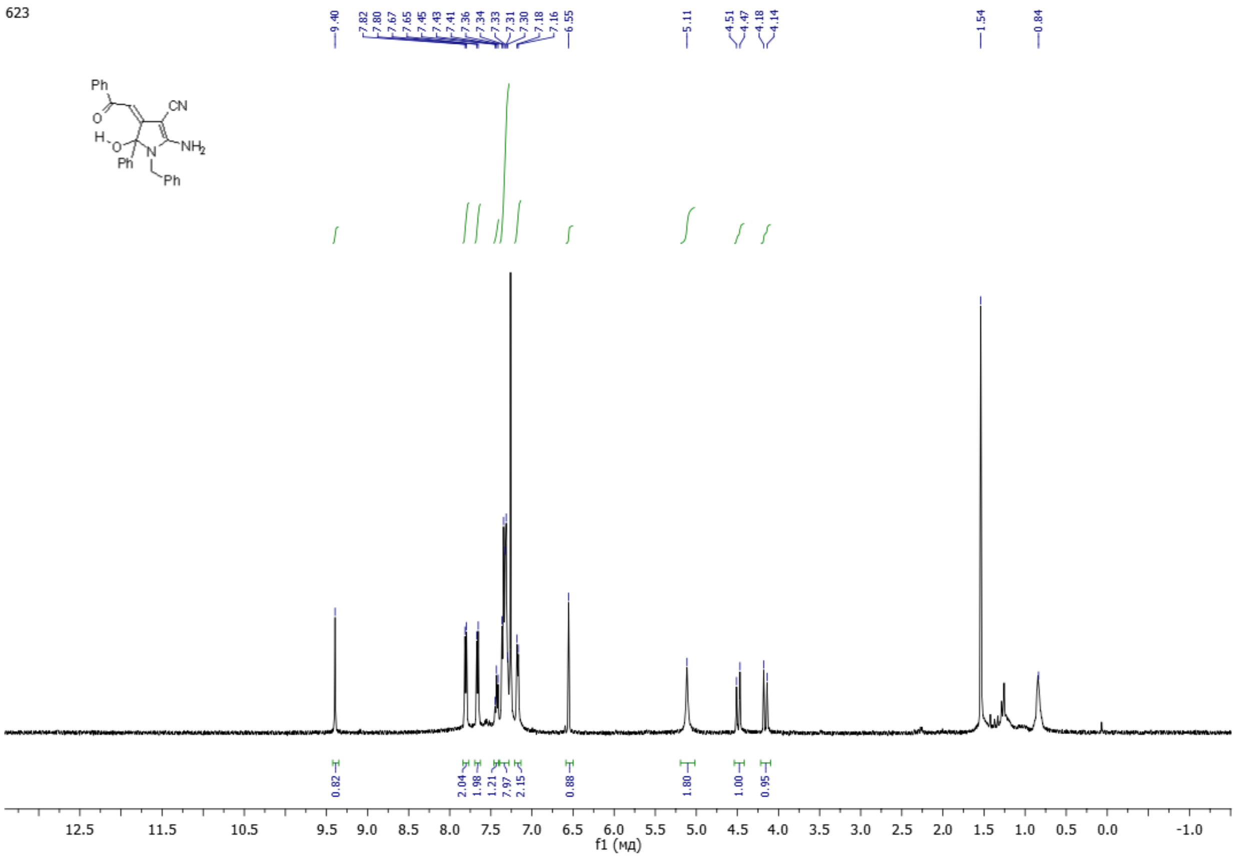

Figure S49 ^{1}H NMR spectrum ($CDCl_3$) of **7b**

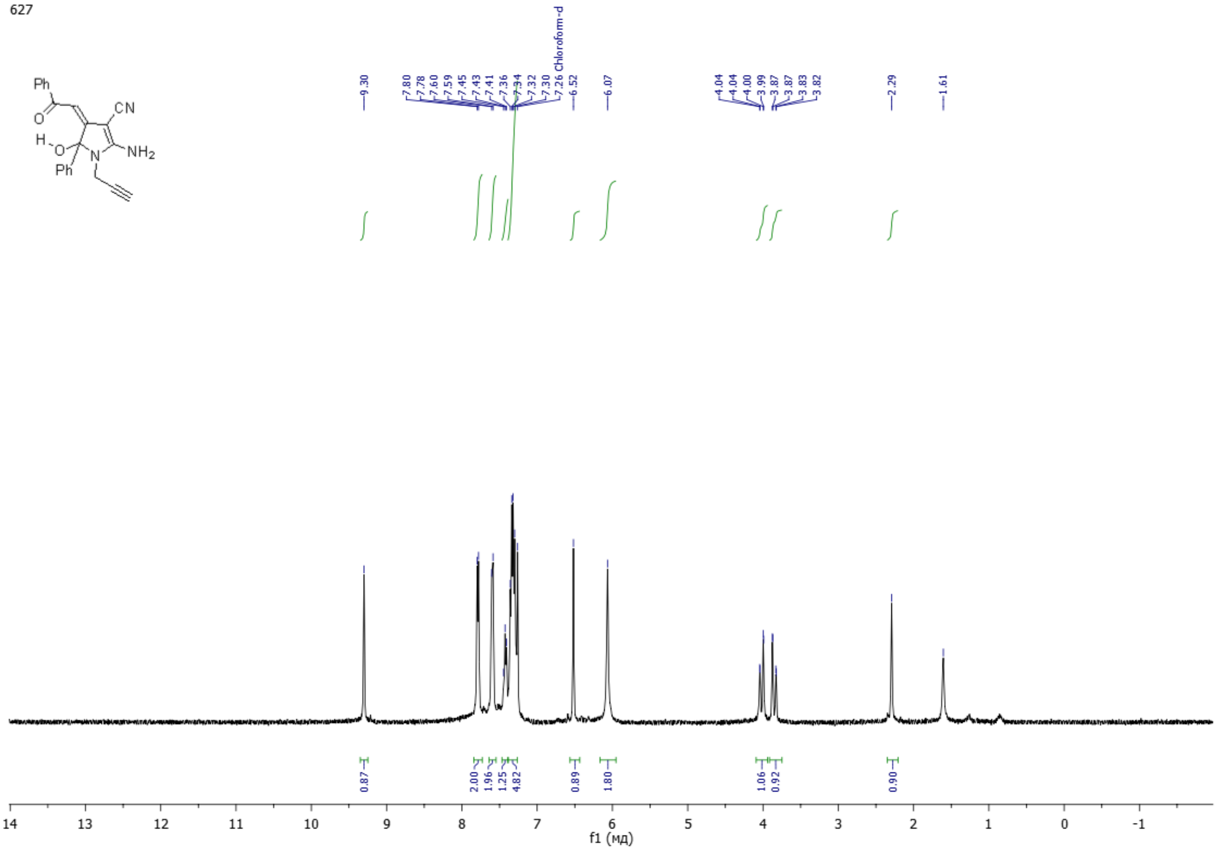

Figure S50 ^{13}C NMR spectrum ($CDCl_3$) of **7b**

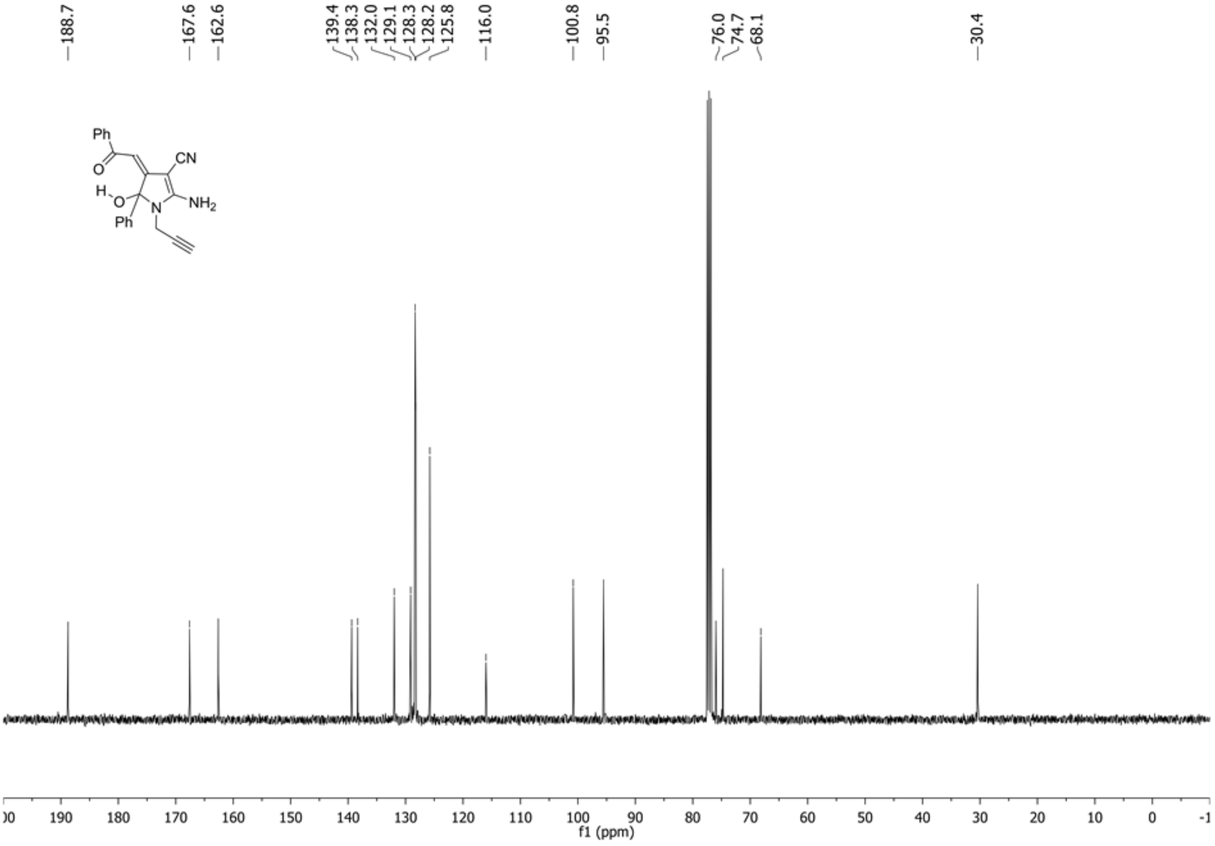

Supplement: Supplementary file 1 [file molecules-28-03576-s001.zip › molecules-2337419-supplementary.pdf]
